# Somite Compartments in Amphioxus and Its Implications on the Evolution of the Vertebrate Skeletal Tissues

**DOI:** 10.3389/fcell.2021.607057

**Published:** 2021-05-10

**Authors:** Luok Wen Yong, Tsai-Ming Lu, Che-Huang Tung, Ruei-Jen Chiou, Kun-Lung Li, Jr-Kai Yu

**Affiliations:** ^1^Institute of Cellular and Organismic Biology, Academia Sinica, Taipei, Taiwan; ^2^Department of Aquatic Biology, Chia-Yi University, Chia-Yi, Taiwan; ^3^Department of Anatomy and Cell Biology, School of Medicine, College of Medicine, Taipei Medical University, Taipei, Taiwan; ^4^Marine Research Station, Institute of Cellular and Organismic Biology, Academia Sinica, Yilan, Taiwan

**Keywords:** cephalochordate, *SPARC*, collagen, skeletal tissue, somite, BMP, evolution

## Abstract

Mineralized skeletal tissues of vertebrates are an evolutionary novelty within the chordate lineage. While the progenitor cells that contribute to vertebrate skeletal tissues are known to have two embryonic origins, the mesoderm and neural crest, the evolutionary origin of their developmental process remains unclear. Using cephalochordate amphioxus as our model, we found that cells at the lateral wall of the amphioxus somite express *SPARC* (a crucial gene for tissue mineralization) and various collagen genes. During development, some of these cells expand medially to surround the axial structures, including the neural tube, notochord and gut, while others expand laterally and ventrally to underlie the epidermis. Eventually these cell populations are found closely associated with the collagenous matrix around the neural tube, notochord, and dorsal aorta, and also with the dense collagen sheets underneath the epidermis. Using known genetic markers for distinct vertebrate somite compartments, we showed that the lateral wall of amphioxus somite likely corresponds to the vertebrate dermomyotome and lateral plate mesoderm. Furthermore, we demonstrated a conserved role for BMP signaling pathway in somite patterning of both amphioxus and vertebrates. These results suggest that compartmentalized somites and their contribution to primitive skeletal tissues are ancient traits that date back to the chordate common ancestor. The finding of *SPARC*-expressing skeletal scaffold in amphioxus further supports previous hypothesis regarding *SPARC* gene family expansion in the elaboration of the vertebrate mineralized skeleton.

## Introduction

Vertebrate skeletal tissues consist of cartilage, bone, dentine and enamel distributed across the dermal skeleton, axial skeleton, and appendicular skeleton (Hall, 2005). Such mineralized skeletal tissues are considered one of the major innovations of vertebrates (Shimeld and Holland, 2000). Among extant animals, only jawed vertebrates possess mineralized skeletal tissues; meanwhile, jawless vertebrates (hagfishes and lampreys) have only non-calcified cartilaginous endoskeletons and lack clear evidence of mineralized skeletal tissues (Zhang and Cohn, 2006; Ota et al., 2011, 2013). Interestingly, however, fossil records show that certain extinct jawless vertebrates did possess mineralized dermal skeletons (e.g., armor plates and scales of the ostracoderms), suggesting that mineralized skeletal tissues may have deep evolutionary origins extending back at least to the vertebrate common ancestor (Donoghue et al., 2006; Janvier, 2015).

Cephalochordates (amphioxus) are invertebrate chordates now considered to be the most basal group within the chordate lineage (Bourlat et al., 2006; Delsuc et al., 2008; Putnam et al., 2008). Therefore, these creatures occupy a key phylogenetic position for understanding the evolution of novel vertebrate characteristics. In the search for a possible evolutionary precursor of skeletal tissue, a recent study revealed that the oral tentacles in amphioxus are cartilage-like structures, as their formation requires FGF signaling, and orthologs of vertebrate cartilage markers are expressed in the developing tissues (Jandzik et al., 2015). These results are consistent with an earlier finding that after amputation, the regenerating amphioxus oral tentacles also express cartilage marker genes and the cartilage/bone matrix protein gene, *SPARC* (Kaneto and Wada, 2011). As such, the histological and molecular features of the amphioxus oral skeleton are similar to those of vertebrate cellular cartilage. In addition to the cartilaginous oral cirri, a dermal skeleton-like collagenous structure has also been described in amphioxus (Mansfield et al., 2015; Yong and Yu, 2016). However, the embryonic origins of amphioxus “skeleton-like” cells have not been thoroughly studied.

In vertebrate embryos, skeletogenic cells are derived from the somitic mesoderm, lateral plate mesoderm, and neural crest. Because amphioxus lacks neural crest cells, it has been hypothesized that embryonic mesoderm is the likely source of progenitor cells that contribute to its cartilage-like tissues (Holland, 2009; Medeiros, 2013; Green et al., 2015). Previously, distinct mesodermal compartments were found in amphioxus, raising the possibility that these compartments may be homologous to the mesodermal domains that give rise to skeletogenic tissues in vertebrates (Gostling and Shimeld, 2003; Onimaru et al., 2011; Mansfield et al., 2015). In vertebrates, developing somites can be divided into three major domains, the lateral dermomyotome, the centromedial myotome, and the ventromedial sclerotome; of note, the sclerotome contributes to the axial skeleton, while the dermomyotome contributes to the dermal skeleton (Scaal and Wiegreffe, 2006). It has been well documented that the medial wall of the amphioxus somite (considered to be its myotome) contributes to the striated musculature (Holland et al., 1995). While the developmental fate of the amphioxus lateral somite was previously obscure, a recent TEM study revealed that during development, the non-myotome somite is compartmentalized into dorsolateral and ventromedial domains, which respectively resemble the vertebrate dermatome and sclerotome; intriguingly, the ventrolateral somite buds out ventrally to form the mesothelia of the perivisceral coelom (Shimeld and Holland, 2000; Gostling and Shimeld, 2003; Scaal and Wiegreffe, 2006; Mansfield et al., 2015). Additionally, cells from the sclerotome-like domain expand medially as a cell sheet to enclose the amphioxus notochord and neural tube, eventually contributing to the formation of connective tissues along axial structures (Mansfield et al., 2015). These observations suggest that certain aspects of somite compartmentalization may predate the origin of vertebrates. However, the degree to which amphioxus somite compartments are similar to major vertebrate somite compartments, in terms of molecular topology and patterning mechanisms, remains unclear. In addition, the questions of whether the amphioxus non-myotome somite derivatives contribute to the formation of skeleton-like structures, and whether these structures may have any trace of tissue mineralization are still unresolved.

To address these questions, we first set out to study the expression pattern of the amphioxus *SPARC* gene, as its vertebrate homologs play crucial functions in tissue mineralization (Bradshaw, 2009). *SPARC* (encoding Secreted Protein Acidic and Rich in Cysteine) is an ancient gene found in both protostomes and deuterostomes (Bertrand et al., 2013). We use the term *SPARC* to refer to *SPARC/SPARCL1* analyzed by Bertrand et al. (2013) for this study. Its vertebrate orthologs are expressed in cartilage, bones, and teeth, where they can bind calcium and act as extracellular collagen chaperones (Kawasaki and Weiss, 2006). More importantly, specific *SPARC* family members have been duplicated in jawed vertebrates to generate the secretory calcium-binding phosphoprotein (SCPP) family of genes, which are involved in the formation of highly-mineralized tissues, such as dentin and enamel (Kawasaki and Weiss, 2003; Kawasaki et al., 2005). Here we show that the lateral amphioxus somite expresses *SPARC* and various collagen genes during embryonic development. Furthermore, the somite-derived *SPARC*-expressing cells are closely associated with the formation of collagenous supporting tissues in juvenile amphioxus, leading to the hypothesis that these collagenous tissues may be homologous to the mineralized skeletal tissues in vertebrates. To test whether the somite developmental program is conserved between amphioxus and vertebrates, we first extensively surveyed a suite of amphioxus transcription factor genes with homologs that mark distinct somite compartments and the lateral plate mesoderm in vertebrate model systems. Then, we performed functional experiments with a small molecule inhibitor and recombinant protein to further test whether amphioxus somites are patterned by conserved signaling mechanisms. Our results suggest an overall conservation of the chordate paraxial mesoderm patterning mechanism and provide important insights into the developmental origin of mesoderm-derived skeletal tissues during chordate evolution.

## Results

### *SPARC* Is Co-expressed With Collagen Genes in the Lateral Somite of Amphioxus

**FIGURE 1:**
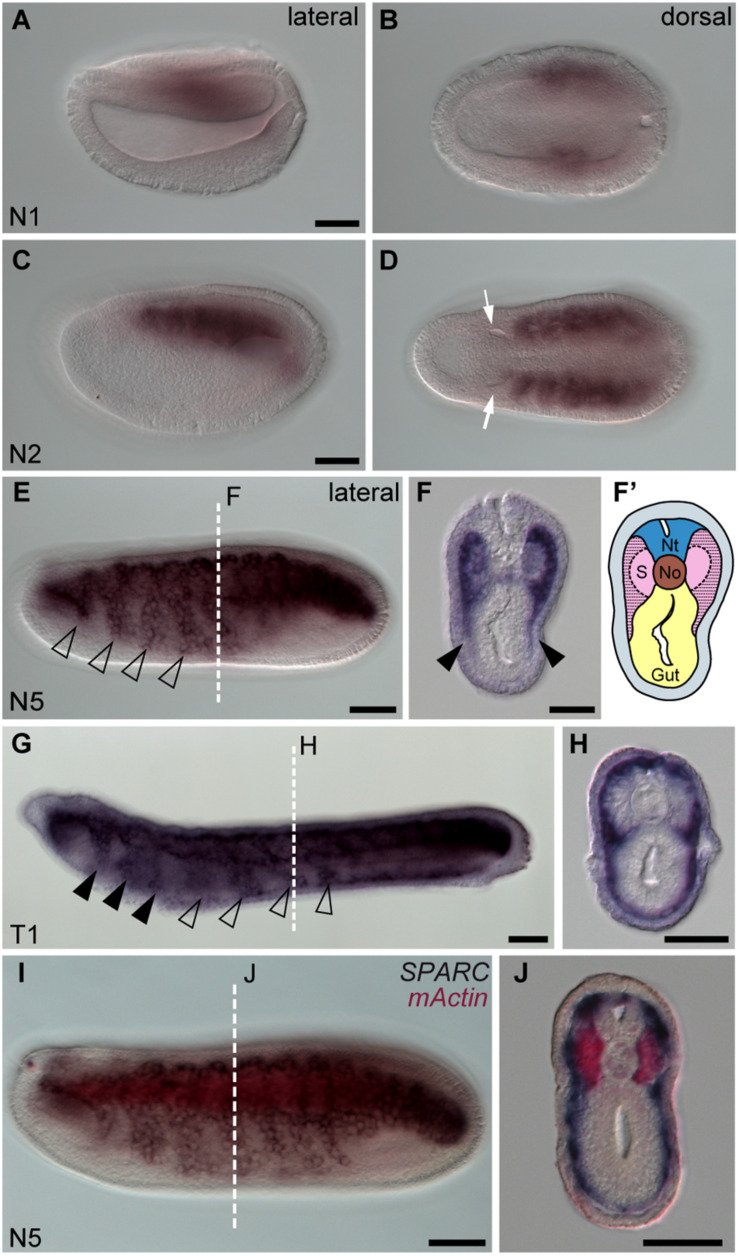
Amphioxus *SPARC* is expressed in the lateral region (non-myotome part) of the somite. Images **(A,C,E,G,I)** were taken from the lateral view; the anterior is to the left and dorsal is to the top. Scale bars, 50 μm. Images **(B,D)** were taken from the dorsal view, where the anterior is to the left and the right side is on top. Scale bars in **(A,C)** apply to **(B,D)**. Cross sections in panels **(F**,**H,J)** show the respective level marked with a white dashed line in panels **(E**,**G,I)**. Scale bars, 25 μm. **(E)** *SPARC* expressing cells expand ventrally (hollow arrowhead). **(F)** Black arrowheads indicate the lateral part of the somite that expresses *SPARC* and extends ventrally. **(F′)** Schematic drawing of panel **(F)**; area filled with dashed lines marks where *SPARC* is expressed. Nt, neural tube; S, somites; No, notochord. **(G)** *SPARC*-expressing cells move to the pharyngeal region (black arrowheads). **(I)** Double *in situ* hybridization of *SPARC* (dark purple) and *mActin* (red) of a late N5 stage embryo.

Using carefully staged amphioxus embryos (Carvalho et al., 2021), we found that *SPARC* was initially expressed in the paraxial mesoderm at the early neurula stage (N1 stage, Figures 1A,B), and then at the N2 stage it was strongly expressed in all the segmented somites, except for the first somite (Figures 1C,D). This pattern is consistent with a previous report (Bertrand et al., 2013). We also noticed that at the subsequent N5 stage, *SPARC* expression was expanded to all developing somites, and its expression was downregulated in the medial part of the somites (Figures 1E,F). Moreover, the lateral region of the somites that expresses *SPARC* started to extend ventrally (Figures 1E,F, arrowheads). By the T1 stage, *SPARC*-positive cells at the rostral level had entered the pharyngeal region (Figure 1G, arrowheads). Meanwhile, at the trunk level, *SPARC*-expressing cells had expanded both laterally and ventrally to form a thin layer beneath the epidermis, and some *SPARC*-expressing cells had extended medially to surround the notochord and neural tube (Figure 1H). The behavior of these *SPARC*-expressing somite derivatives is in accordance with a previous report on the development of non-myotome somite cells (Mansfield et al., 2015). The locations of the medial (myotome) and lateral (non-myotome) regions of the amphioxus somite were further confirmed by double *in situ* hybridization with muscle actin gene (*mActin*) and *SPARC*, which revealed that the two genes were expressed in distinct domains (Figures 1I,J). The medial somite cells fated to differentiate into the myotome were clearly labeled by *mActin* expression, while *SPARC* expression marked the lateral non-myotome somite cells that would eventually expand ventrally and medially (Figure 1J).

**FIGURE 2:**
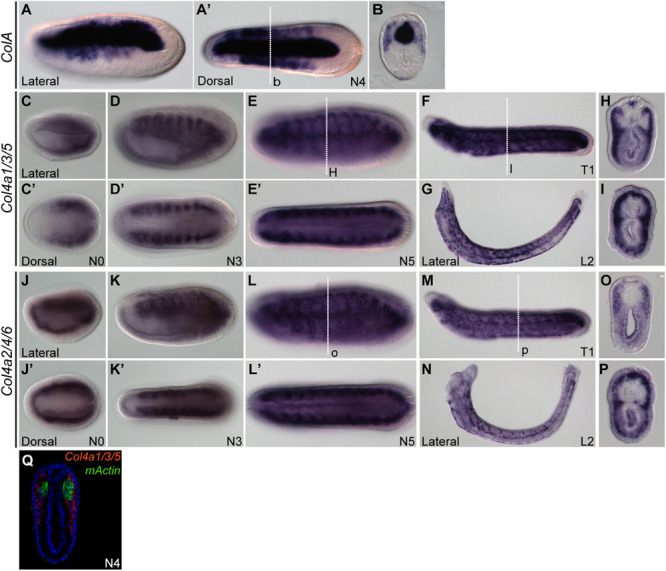
Expression domains of amphioxus CollagenA (*ColA*) and CollagenIV (*Col4*) genes. **(A,B)** Expression pattern of *ColA* **(A,A′)** N4 stage embryo, lateral and dorsal views. **(B)** Cross section of N4 embryo in panel **(A′)** at the level marked with a white dashed line. **(C–I)** Expression pattern of *Col4a1/3/5*. **(C,C′)** N0 stage embryo, lateral and dorsal view. **(D,D′)** N3 stage embryo, lateral and dorsal view. **(E,E′)** N5 stage embryo, lateral and dorsal view. **(F)** T1 stage larva, lateral view. **(G)** L2 stage larva, lateral view. **(H)** Cross section of the N5 embryo in panel **(E)** at the level marked with a white dashed line. **(I)** Cross section of the T1 larva in panel **(F)** at the level marked with a white dashed line. **(J–P)** Expression pattern of *Col4a2/4/6*. **(J,J′)** N0 stage embryo, lateral and dorsal view. **(K,K′)** N3 stage embryo, lateral and dorsal view; *Col4a2/4/6* is expressed in the lateral part of the somite and minimally in the endoderm. **(L,L′)** N5 stage embryo, lateral and dorsal view. **(M)** T1 stage larva, lateral view. **(N)** L2 stage larva, lateral view. **(O)** Cross section of the N5 stage embryo in panel **(L)** at the level marked with a white dashed line. **(P)** Cross section of T1 stage larva in panel **(M)** at the level marked with a white dashed line. **(Q)** Cross section of N4 stage double FISH of *Col4a1/3/5* with *mActin*.

Previously, it was reported that amphioxus fibrillar collagen gene (*ColA*), which represents the sole homolog of vertebrate collagen I, II, III, and V genes, is expressed in non-myotome somite derivatives, and in the notochord and neural tube (Meulemans and Bronner-Fraser, 2007; Wada, 2010; Mansfield et al., 2015). We found that at the N4 stage, the expression pattern of *ColA* in the lateral portion of the developing somites was similar to that of *SPARC* (Figures 2A,A’,B), which clearly marked the non-myotome somite cells expanding laterally and ventrally. We further examined the expression of amphioxus collagen IV genes (*Col4a1/3/5* and *Col4a2/4/6*), the gene products of which are the most abundant collagen proteins in the basement membrane (Sasaki et al., 1998; Bradshaw, 2009). Both genes were co-expressed with *SPARC* in the non-myotome region of the somite and its derivatives (Figures 2C–Q). Together, these results indicate that *SPARC* is co-expressed with multiple collagen genes in the non-myotome somite and its derivatives.

**FIGURE 3:**
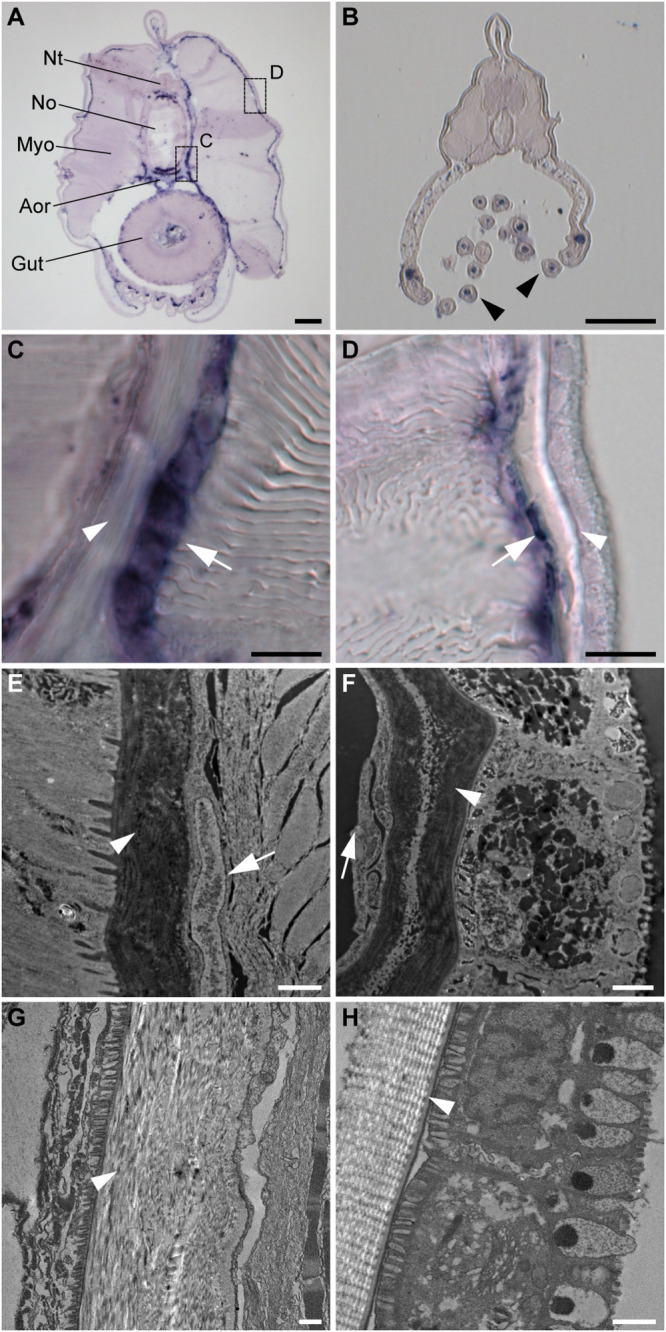
Amphioxus *SPARC* is expressed in the mesothelial layer during advanced developmental stages. **(A)** Cross section of a juvenile amphioxus. Nt, Neural tube; No, Notochord; Aor, Aorta; Myo, Myotome; Scale bar, 50 μm. **(B)** Cross section of a juvenile amphioxus, taken near the rostral region. Black arrowheads mark oral tentacles. Scale bar, 50 μm. **(C)** Magnification of the area around the notochord region, indicated by the dashed box “c” in panel **(A)**. White arrows indicate the *SPARC*-expressing cells. White arrowhead indicates collagenous structures. Scale bar, 10 μm. **(D)** Magnification of the area around the epidermal region indicated by the dashed box “d” in panel **(A)**. White arrows indicate *SPARC*-expressing cells. White arrowhead indicates collagenous structures. Scale bar, 10 μm. **(E,F)** TEM images of **(E)** the notochord and **(F)** the epidermis of a young juvenile. White arrows indicate putative *SPARC*-expressing mesothelial cells. White arrowheads indicate collagenous structures. Scale bar, 1 μm. **(G,H)** TEM images of **(G)** the notochordal sheath and **(H)** the epidermis of an older juvenile roughly corresponding to images **(E)** and **(F)**, respectively. White arrowheads mark collagenous structures. Scale bar, 1 μm.

To further characterize the non-myotome somite derivatives, we next analyzed the *SPARC*-expressing cells in juvenile amphioxus and found that the cells are present around the notochord and neural tube, and underneath the epidermis (Figure 3A). Comparing higher magnifications (Figures 3C,D) and TEM images (Figures 3E,F) to previously published data, we found the *SPARC*-expressing cells were very similar to previously described mesothelial cells (Ruppert, 1997; Mansfield et al., 2015). In addition, we found that *SPARC*-expressing cells were present in the developing oral tentacles (Figure 3B), which represent the oropharyngeal skeleton in amphioxus and are akin to vertebrate cellular cartilage (Jandzik et al., 2015). Importantly, we noted that amphioxus *SPARC*-expressing cells are often found adjacent to dense collagenous mesh structures, including those around the notochord and underneath the epidermis (Figures 3C–H), suggesting that the cells may contribute to the formation of extracellular collagen connective tissues at these locations.

### Amphioxus Somites Contain Mesodermal Compartments That Are Comparable to Vertebrate Somites and Lateral Plate Mesoderm

**FIGURE 4:**
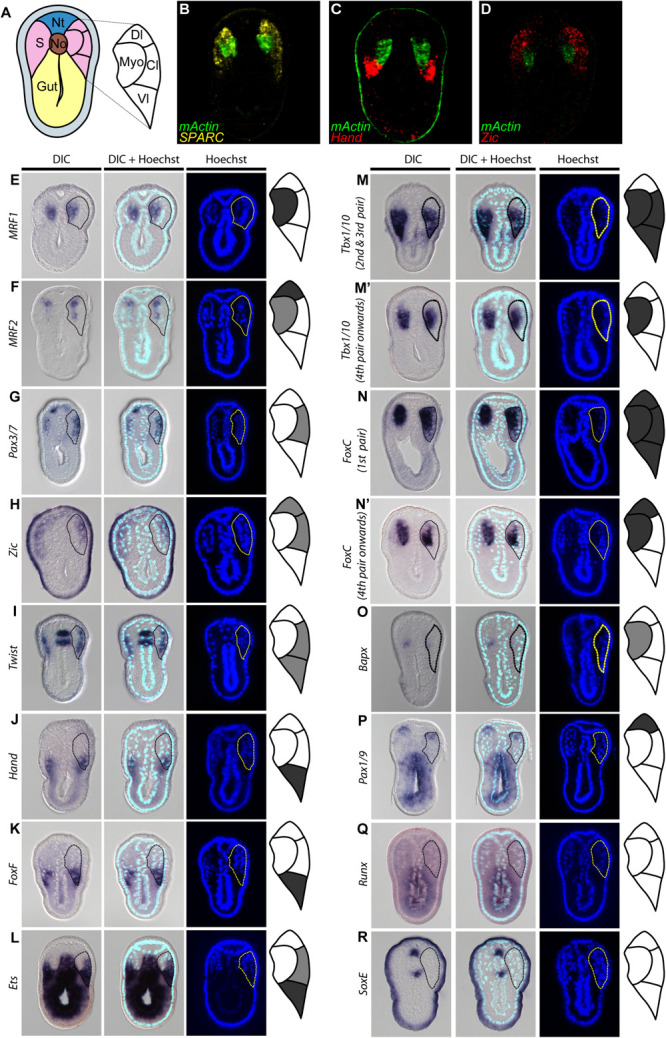
Comparable compartments inside the amphioxus somite can be recognized by the expression of specific marker genes. **(A)** Schematic drawing of an N3 stage (six somite) embryo cross section. The somite region (pink) can be compartmentalized into four regions based on the expression of different marker genes. Myo, myotome; Dl, dorsolateral; Cl, Centrolateral; Vl, ventrolateral; Nt, neural tube; S, somite; No, Notochord. **(B–D)** Double fluorescent *in situ* hybridization of sectioned embryos. **(E–R)** Expression pattern of known somite marker genes in N3 stage embryos, taken from around 5th somite level. In the schematic diagrams, dark gray represents stronger expression while light gray represents weaker expression. The right somite is outlined with dashed lines. **(Q,R)** Expression patterns of known vertebrate sclerotome marker genes that are not expressed in the amphioxus somites. The right somite is outlined with dashed-lines.

Our observations of *SPARC* and collagen gene expression raised a question of whether the amphioxus somite is compartmentalized by molecular mechanisms homologous to those that pattern the vertebrate somite. To address this question, we extensively surveyed the expression patterns of amphioxus homologs of vertebrate genes known to mark distinct somite compartments. We focused on the N3 stage (with 6 somite pairs), when the most ventral part of the amphioxus somite begins to grow downward (Figure 4). We used multi-color fluorescence *in situ* hybridization to delineate the exact expression domains and boundaries of individual genes in the same image, allowing us to define distinct somite compartments in amphioxus (Figures 4A–D and Supplementary Figure 1). Based on gene expression profiles, we delineated four distinct compartments in the developing amphioxus somite (Figure 4A). A myotome domain was defined based on the expression of *mActin* and *MRF1* (Figures 4B,E). In contrast to a previous publication, we found *MRF2* was expressed in the dorsolateral domain of the somite as well as medial domain (Figure 4F; Schubert et al., 2003). Homologs of vertebrate dermomyotome markers, *Pax3/7*, *Zic*, and *Twist*, were expressed in the lateral somite (Figures 4D,G–I); *Zic* was absent from the most ventrolateral region of the somite (Figures 4D,H), and *Twist* expression was absent from the most dorsolateral region (Figure 4I). We also found that *Hand*, *FoxF*, and *Ets* (homologs of vertebrate lateral plate mesoderm markers) were expressed in the ventrolateral region of the somite (Figures 4J–L), with *Ets* expression extending dorsally to the centrolateral somite (Figure 4L). Interestingly, amphioxus homologs of sclerotome markers *Tbx1/10*, *FoxC*, and *Bapx* (*Nkx3*) were expressed in the medial part of the somite that overlaps with the presumptive myotome domain (Figures 4M–O). *Tbx1/10* was also expressed in the centrolateral and ventrolateral somite domains, consistent with a previous report (Mahadevan et al., 2004). At the 2nd and 3rd somite pair, *Tbx1/10* expression marked the ventral somite outgrowth (Figure 4M and Supplementary Figure 2A shows N5 stage). On the other hand, *Pax1/9* was expressed in the dorsolateral domain of the somite, while its homologs in amniotes were expressed in the ventromedial somite, marking the developing sclerotome (Rodrigo et al., 2003). Moreover, we found that some homologs of vertebrate sclerotome markers, including *Runx* and *SoxE*, were not expressed in amphioxus somites (Figures 4Q,R), indicating that although distinct somite compartments can be recognized, amphioxus may lack certain key genetic toolkits that define the sclerotome compartment. Our results thus indicate that the amphioxus somite is compartmentalized into regions equivalent to vertebrate dermomyotome, myotome, and lateral plate mesoderm, with sclerotome markers present in the medial portion of the somite.

### BMP Signaling Pathway Controls the Medial-Lateral Patterning of Amphioxus Somites

**FIGURE 5:**
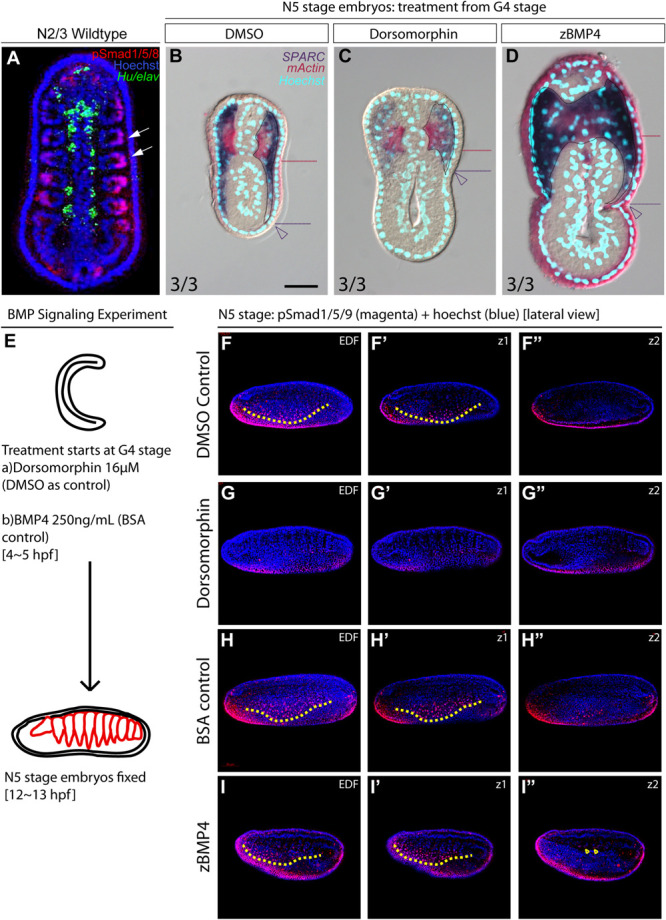
Medial-lateral patterning of amphioxus somites is controlled by the level of BMP signaling. **(A)** Dorsal view of a stage N2/3 embryo. Anterior is to the top. Nuclear signals of pSmad1/5/8 are present preferentially in the lateral part of the somites (white arrows). *Hu/elav* (green) label indicates the midline structure in the embryo. **(B–D)** Cross sections of N5 embryos treated with BMP signaling inhibitor Dorsomorphin or recombinant zBMP4 protein at G4 stage. Scale bar, 25 μm. Red dotted lines mark the ventral boundary of the myotome region. Purple dotted lines mark the ventral boundary of the lateral somite region. Numbers on bottom left: denominator represents the total number of embryos that sectioned at 4∼6th somite level, numerator shows the number of embryos which show the displayed phenotype when sectioned. DMSO control is used as the count number for the control column. BSA whole mount embryos are shown in Supplementary Figure 4. **(B)** Double *in situ* hybridization showing the expression domain of *SPARC* and *mActin* in a control embryo treated with DMSO. **(C)** Section of N5 embryo treated with 16 μM dorsomorphin. **(D)** Section of N5 embryo treated with 250 ng/ml of zBMP4 protein. **(B–D)** Whole mount embryo of cross section in panels **(B–D)** is shown in Supplementary Figure 4. **(E)** Treatment time and concentration of BMP Signaling experiment. **(F–I″)** pSmad1/5/9 staining of embryos after BMP Signaling perturbation. Lateral view. Yellow dotted lines demarcate the ventral limit of the pSmad1/5/9 nuclear signal in the ventrally expanding somite. EDF, Extended Depth of Focus. Z1 demarcates the z-level which that corresponds to the EDF view. Z2 demarcates the z-level that is approximate at the notochordal level. **(I″)** Yellow arrowheads point to positive pSmad1/5/9 nuclear signal which roughly corresponds to the region where lateral gene markers are ectopically disorganized.

**FIGURE 6:**
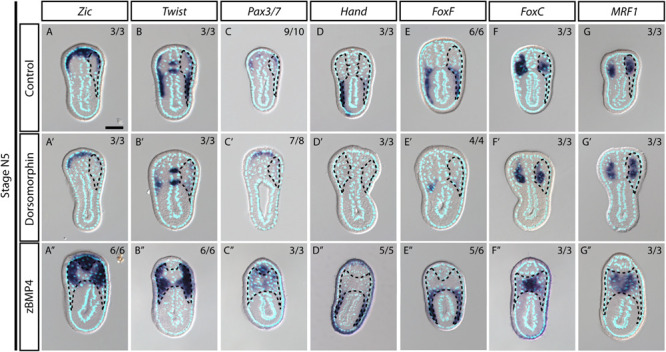
Medial-lateral patterning of amphioxus somites is controlled by the level of BMP signaling. **(A–G)** Cross sections of N5 stage embryoes treated with DMSO as a control group. Scale Bar, 25 μm. **(A′–G″)** Cross sections of N4 stage embryos treated with inhibitor Dorsomorphin or recombinant zBMP4 protein at G4 stage. Dotted black outline marks one side of the somite. Whole somite areas are marked in panels **(A″–G″)**, as left and right somites are not readily discernible. Left and right sides of somites exhibit left-right asymmetry as the left amphioxus somite approximately half a segment staggered more anteriorly to the right somite. Numbers on top right: denominator represents the total number of embryos that are sectioned at approximately 6th to 8th somite level as shown in Supplementary Figure 2, numerator shows the number of embryos which show the displayed phenotype when sectioned. DMSO control is used as the count number for the control column. BSA treated whole mount embryos are shown in Supplementary Figures 5–8. Whole mount embryos used from experiment are displayed in Supplementary Figures 5–8, sections are obtained from 6th to 8th pair somite, as indicated by the white brackets drawn in Supplementary Figure 2.

During vertebrate somite development, interactions between several signaling pathways are required to specify different somite compartments (Brand-Saberi and Christ, 2000). One such pathway, BMP signaling, participates in the patterning of the paraxial mesoderm and somitic compartments (Pourquié et al., 1996; Tonegawa et al., 1997). We observed high phospho-Smad1/5/8 nuclear signals in the lateral part of the amphioxus somite during development (Figure 5A, white arrows), indicating that BMP signaling is high in this region. This observation raised the possibility that the patterning function of BMP signaling may be conserved in the amphioxus somite. To investigate this possibility, we manipulated the BMP signaling level during development by treating amphioxus embryos with recombinant BMP4 protein or Dorsomorphin, a small molecule BMP signaling inhibitor. We observed that pSmad1/5/9 nuclear signals in the somite are diminished upon inhibition with Dorsomorphin compared to the controls (Figures 5F–G″ and Supplementary Figures 10F–F″). With zBMP treated embryos, pSmad1/5/9 is maintained in the ventral portion of the somite (Figures 5H–I″) but ecotopic pSmad1/5/9 nuclear signal can be found concentrated in the more medial portion of the somite (Figure 5I″, yellow arrowheads and Supplementary Figures 10I,I″). In Dorsomorphin-treated embryos, expression of lateral marker genes was greatly reduced, and the non-myotome domain did not expand ventrally (Figures 5B,C), while gene expression in the median myotome domain remained largely unchanged. Consistently, expression of other marker genes, including *Zic*, *Twist*, and *Pax3/7* in the lateral domain was greatly reduced (Figures 6A′–C′), and expression of *Hand* and *FoxF* in the ventral lateral domain was also greatly diminished (Figures 6D′–E′). Expression of medial domain markers, such as *FoxC* and *MRF1*, was not affected by Dorsomorphin treatment (Figures 6F′–G′). *Tbx1/10*, which exhibit dynamic expression along the AP axis (Supplementary Figure 2), were greatly downregulated in the ventrolateral portion of the second and third somite pairs, while their expression in the medial domain of the other somites was not affected.

In contrast to the BMP4 inhibitor-treated embryos, those treated withzBMP4 had medially expanded non-myotome domains (Figures 5B,D), while exhibiting variable degree of ventral extension of the lateral somite compared to the control embryos. Additionally, the myotomal domain appeared to merge at the midline (Figure 5D). After zBMP4 treatment, the expression domain of *Zic* and *Twist* was broadened compared to that in controls (Figures 6A″–B″). The *Pax3/7* expression domain expanded medially and merged in the midline (Figure 6C″). Expression of *Hand* was not affected, while *FoxF* expression was expanded more medially (Figures 6D″–E″). *FoxC* and *MRF1* expression merged in the center of the embryo (Figures 6F″–G″). The lateral somite domain of *Tbx1/10* exhibited upregulated expression, while expression in the medial domain merged in the center, similar to the other myotome marker genes (Supplementary Figure 2). We noticed that in zBMP4 treated embryos the ventral expansion of lateral somite appeared to vary among individual embryos (Figures 5, 6 and Supplementary Figures 6–8). Nevertheless, our results provide clear evidence that medial-lateral patterning of amphioxus somite compartments is controlled by BMP signaling levels.

## Discussion

We found that in the cephalochordate amphioxus, the non-myotome somite expresses a conserved tissue-mineralization gene, *SPARC*, along with several collagen genes. The derivatives of this somite region likely contribute to the population of mesothelial cells that generate collagen-based supporting tissues in adult amphioxus (Ruppert, 1997; Mansfield et al., 2015). Furthermore, our gene expression survey of N3 stage (six somite pairs) amphioxus embryos revealed that the initial enterocoelic somites of amphioxus already possess mesodermal progenitor lineages that are comparable to the somitic mesoderm and lateral plate mesoderm in vertebrates. In addition, our functional experiments confirmed the involvement of BMP signaling in medial-lateral patterning of amphioxus trunk somites, which is similar to the patterning mechanism reported in vertebrate model systems (Pourquié et al., 1996; Tonegawa et al., 1997). We use the term medial-lateral patterning in conjunction to the term used in chick embryology (Pourquié et al., 1996; Tonegawa et al., 1997). Our somite orientation also correspond to dorso-ventral somite patterning in anamniotes, which is comparable to amniote medial-lateral orientation (Scaal and Wiegreffe, 2006). Taken together, our results suggest an overall conservation of chordate mesoderm patterning mechanisms. The findings provide important insights into how somite compartmentalization in the chordate lineage may have evolved and enable us to propose a plausible scenario for the developmental evolution of vertebrate mineralized skeletal tissues from somite derivatives.

### Compartmentalized Somites Evolved in the Common Ancestors of All Chordates

Vertebrate somites are compartmentalized into the dermomyotome, myotome, and sclerotome, which respectively give rise to the dermis, skeletal muscle and skeletal elements, among other tissues (Christ and Ordahl, 1995; Gros et al., 2005; Relaix et al., 2005; Christ et al., 2007). In anamniotes, the majority of each somite consists of myotome, and amniote somites are composed by a more prominent sclerotome (Scaal and Wiegreffe, 2006). Among jawless vertebrates, lamprey possess a more anamniote-like somite (with thin sclerotome), while hagfish exhibit a more amniote-like sclerotome (Ota et al., 2011). Despite these differences, the somites of most vertebrates display a recognizable morphological archetype, which implies the interspecific homology of these structures.

**FIGURE 7:**
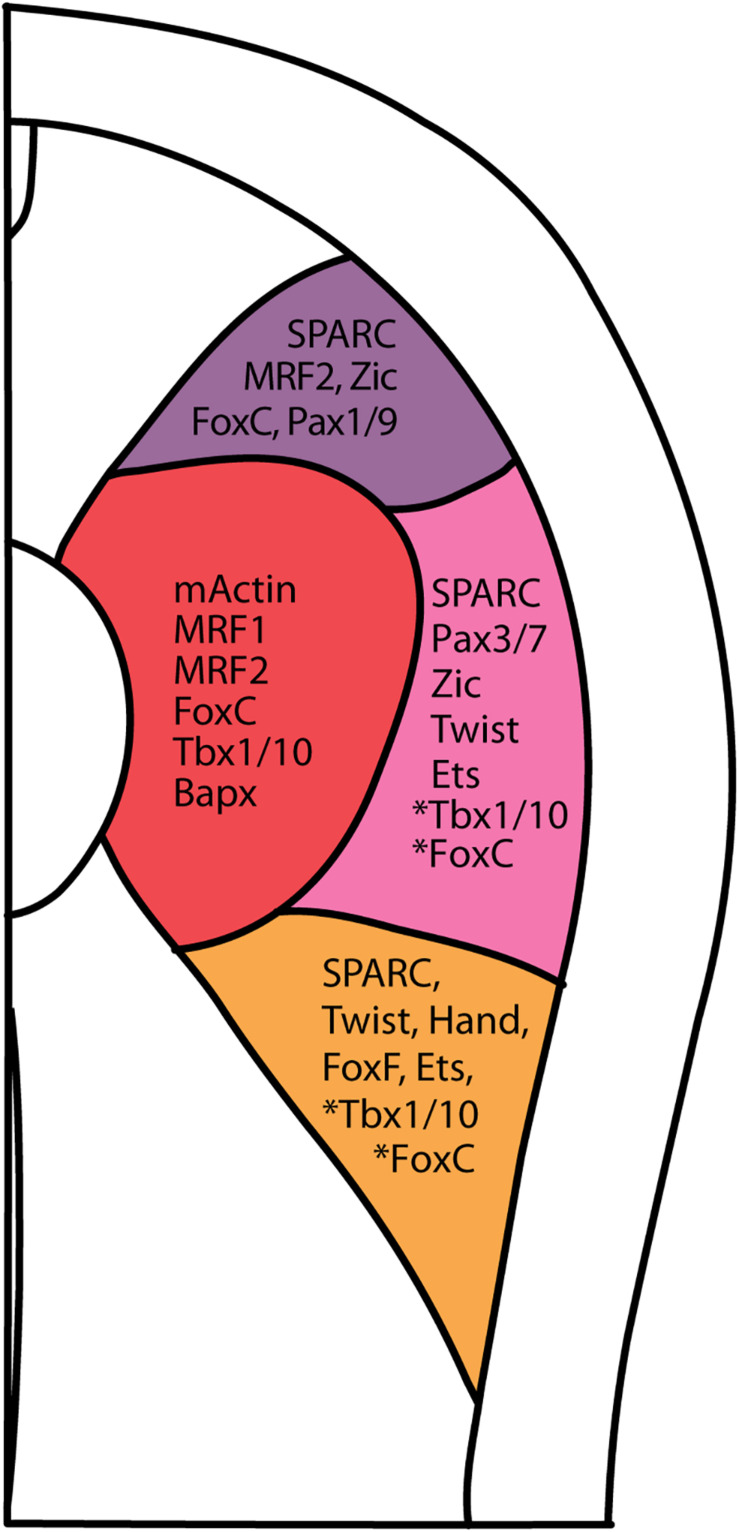
Summary of vertebrate somite compartment markers in amphioxus at approximately 5th somite level of a N3 stage embryo. Asterisk next to *Tbx1/10* and *FoxC* indicates that the presence of *Tbx1/10* and *FoxC* in centro-lateral and ventro-lateral domain only in 1st somite pair (*FoxC*), and 2nd and 3rd pair somite (*Tbx1/10*).

Amphioxus also possesses a metameric somite that forms by a segmentation mechanism conserved with vertebrates (Beaster-Jones et al., 2008; Onai et al., 2015). It has been shown that formation of the first three pairs of amphioxus somites is FGF signaling-dependent; these anterior somites have been linked to the head mesoderm, whereas the remaining pairs are more akin to the trunk paraxial mesoderm, which forms by an FGF-independent mechanism. This consistency enables us to further characterize the compartments within each of the proposed-trunk somites in amphioxus (Bertrand et al., 2011; Aldea et al., 2019). Here, our compilation of expression patterns of known vertebrate somite marker homologs at a single stage (N3 Stage, six-somite neurula) further strengthens the claim from previous studies that the epithelial cells lateral to the myotome have a dermomyotome, sclerotome and lateral plate mesoderm fate (Figure 7; Gostling and Shimeld, 2003; Onimaru et al., 2011; Mansfield et al., 2015; Aldea et al., 2019).

We show that *Pax3/7*, the amphioxus homolog of the defining vertebrate dermomyotome markers, *Pax3* and *Pax7*, is expressed in the lateral portion of the somite. Since *Pax3/7* is known to be expressed in the dermomyotome of most vertebrates (e.g., cyclostomes, teleosts, and amphibians), we can conclude that the dermomyotome is a conserved feature of the chordate clade (Kusakabe and Kuratani, 2005; Devoto et al., 2006; Epperlein et al., 2007; Hammond et al., 2007; Stellabotte et al., 2007; Ota et al., 2011) (Summarized in Supplementary Table 2). Additionally, the presence of *Zic* and *Twist* expression in the lateral portion of the amphioxus somite provides further evidence for the plesiomorphy of the chordate dermomyotome (Gostling and Shimeld, 2003; Meulemans and Bronner-Fraser, 2007; Supplementary Table 2). In mouse cell culture, *Zic* and *Pax3* work synergistically to activate *Myf5*, an upstream muscle transcription factor (Himeda et al., 2013). *Twist*, a homolog of the vertebrate *Twist1* and *Twist2* (also known as *Dermo*), marks a central dermomyotome-like region in the amphioxus somite. In chicks, such cells give rise to the mesenchymal dermis, a derivative of the dorsal dermomyotome (Scaal et al., 2001). Moreover, ectopic *Twist2* expression in chick dermomyotome causes the formation of dense dermis (Hornik et al., 2005). In trout, *Dermo1* is also expressed in the lateral somatic cells now known as the dermomyotome (Dumont et al., 2008). We therefore conclude that the molecular signatures of a vertebrate dermomyotome is present in a primitive dermomyotome-like structure in amphioxus. Thus, a conserved dermomyotome-like domain in the somite is present throughout the chordate clade. Interestingly, Arthropods have a *Pax7*-expressing satellite cell (resembling a dermomyotome derivative in vertebrates) that contributes to muscle regeneration (Konstantinides and Averof, 2014). Further investigations in other clades of metazoan species will further elucidate the evolutionary trajectory of the dermomyotome.

In chicks, the primary wave for myotome formation emanates from the edges of the epithelial dermomyotome (Gros et al., 2004). The medial edge neighboring the neural tube, called the dorsomedial lip of the dermomyotome (DML), translocates to form the epaxial muscle (Huang and Christ, 2000; Gros et al., 2004). In anamniotes, the formation of the dermomyotome, which subsequently contributes to the myotome, is preceded by the formation of a first wave of muscle precursors from the paraxial mesoderm, prior to somite formation (reviewed in Buckingham and Vincent, 2009; Della Gaspera et al., 2012; Sabillo et al., 2016; Keenan and Currie, 2019). In zebrafish, the Pax3- and Pax7-positive dermomyotome contributes to the medial fast muscle (Hammond et al., 2007). In *Xenopus*, the *Pax3-* and *Meox*-expressing dermomyotome also contributes myogenic cells to the existing median myotome domain (Grimaldi et al., 2004; Della Gaspera et al., 2013). While the myotome region within amphioxus is well established, whether the lateral portion of the somite exhibits a DML-like region and cell movement is less clear (Holland et al., 1995). Both *MRF1* and *MRF2* are expressed in the designated myotome region well before segmentation, indicating an anamniote-like primary myotome-like area (Schubert et al., 2003). Later during development, *MRF2* is also found in the dorsolateral somite, adjacent to dermomyotome markers like *Pax3/7* (Figures 4F,G). This pattern corresponds to the expression of the newly identified amphioxus *MRF4*, which is also found in the dorsolateral portion of the lateral somite (Aase-Remedios et al., 2020). While these expression data suggest a possible DML-like location, further lineage tracing experiments will be needed to determine whether this region behaves like the DML and contributes to myotome precursors. Absence of *Sim* expression in the somite indicates a lack of a vertebrate-like ventromedial lip (VML) region in amphioxus (Coumailleau and Duprez, 2009; Li et al., 2014). Regardless of these intriguing questions, our data suggest that the major players of dermomyotome development were already established in ancestral chordate somites.

The sclerotome is considered to be a vertebrate innovation that gives rise to the axial skeleton (vertebrae) and part of the dorsal aorta (Shimeld and Holland, 2000; Christ et al., 2004; Wiegreffe et al., 2007). A recent study in amphioxus showed that the ventromedial part of the lateral somite also grows medially, sandwiched by the myotome and gut, during the late neurula stage; it later grows dorsally during 4–5 gill slit stage to encase the axial structures (Mansfield et al., 2015). The authors of this previous study considered the more medial mesothelial layer surrounding the axial structure to be a sclerotome-equivalent structure (Mansfield et al., 2015). It should be noted that the study observes closely staged fixed samples. Lineage tracing studies will be needed to further confirm if the medially expanding mesothelial cells indeed originate from the lateral portion of the somite. While a *bona fide* amniote-like sclerotome region cannot be ascribed during amphioxus embryogenesis, amphioxus homologs of known vertebrate sclerotome markers, *Bapx* (*Nk3*), *FoxC*, and *Tbx1/10*, are expressed in the myotome (Chapman et al., 1996; Winnier et al., 1997; Hiemisch et al., 1998; Tanaka et al., 1999). Hence, in amphioxus, the medial portion (be it the myotome or the lateral somite) displays the molecular features of both sclerotome and myotome, reminiscent of the posterior ventromedial cells of teleosts, which give rise to both sclerotome and muscle cells (Morin-Kensicki and Eisen, 1997). In addition, two other important sclerotome markers, *Pax1/9* and *Twist*, are expressed in the dorsolateral and centrolateral portions of the somite, reminiscent of the recently identified dorsal sclerotome domain in teleosts (Mansfield et al., 2015; Ma et al., 2018). The presence of sclerotomal markers in the lateral portion of the somite also corroborates a recent study identifying a multipotent lateral somitic frontier in *Xenopus*, which give rise to both the dermomyotome and sclerotome (Della Gaspera et al., 2019). Together with these previous studies, our data suggest that the somites in the common ancestor of all chordates may have possessed a few rudimentary traits of the vertebrate sclerotome. The *bona fide* vertebrate sclerotome may have evolved as a piecemeal construction of gradually co-opted regulatory toolkits, such as *Runx* and *SoxE*, that are crucial for vertebrate osteogenesis and chondrogenesis (Meulemans and Bronner-Fraser, 2007; Zhang, 2009; Fisher and Franz-Odendaal, 2012; Gómez-Picos and Eames, 2015).

In this study, we also show that a lateral plate mesoderm-like progenitor exists within the lateral compartment of the amphioxus somite. Unlike chicks where the presumptive lateral plate mesoderm is specified before the formation of the segmented somite, amphioxus lateral plate mesoderm-like progenitors are segmented and found within bounds of the ventrolateral portion of the somite (Holland, 2018; Prummel et al., 2019). Expression patterns of *SPARC* at fine time points substantiate that the ventrolateral portion of the somite develops continuously from the lateral somite. This model is consistent with paraxial mesoderm development of most anamniotes, including lamprey and axolotl (Tulenko et al., 2013). Expression of amphioxus *FoxF* and *Hand*, known lateral plate mesoderm markers in vertebrates, during N4/5 stage neurula (9–10 somites) is present in the ventrolateral region of the somite (Onimaru et al., 2011). These results suggest that the lateral plate mesoderm and somite of vertebrates were derived from a common progenitor within an ancestral chordate somite, and subsequent elaborations further lateralized it into the somite, nephridium, and lateral plate mesoderm.

Also, the lateral plate mesoderm is the main embryonic source of the circulatory system (reviewed by Sato, 2013). *Ets*, an important marker for vascular formation, is also expressed in the proposed lateral plate mesoderm region in amphioxus (Meulemans and Bronner-Fraser, 2007; Ellett et al., 2009). This corroborates with the previous identification of cardiogenesis markers (*Csx*, *Hand*, *Tbx4/5*) and hematopoiesis marker (*Pdvegfr*, *Scl*, *GATA1/2/3*) in the currently proposed lateral plate mesoderm like region of the somite (Pascual-Anaya et al., 2013). It should be noted that *Scl* and *Pdvegfr* also overlaps with the proposed nephrogenic region roughly corresponding to the first 3 somite during neurula and early larval stages while *Pdvegfr* is also expressed in the ventrolateral portion of the *SPARC* positive region during L2 stage embryos. In *Drosophila*, *SPARC*-positive hemocyte is required for proper formation of the *Collagen IV*-positive basal lamina (Martinek et al., 2002, 2008). Overall, comparisons of the circulatory system in amphioxus may thus provide further information on the evolution of circulatory systems in metazoa.

In summary, our comprehensive dataset on the molecular identities of somite compartments show that the amphioxus somite shares a consistent general architecture with the vertebrate non-axial mesoderm including the somite and lateral plate mesoderm (Figure 7). It should be noted that the tunicates secondarily lost segmented somites, and its primary muscles are derived from lineage-specific progenitors (Conklin, 1905; Ruppert, 2005). Thus, the amphioxus somite may represent the ancestral form of the chordate somite.

### Conserved BMP Signaling Pathway Controls Medial-Lateral Patterning of the Amphioxus Somite

The compartmentalization of the vertebrate somite is influenced by the proximity of cells to different signaling centers, such as the neural tube, notochord, lateral plate mesoderm, and overlying ectoderm (reviewed in Yusuf and Brand-Saberi, 2006). In chicks, BMP4 from the lateral plate mesoderm was initially found to be crucial for medial-lateral patterning of the paraxial mesoderm (Pourquié et al., 1996). Overexpression of BMP4 between the neural tube and DML causes the lateralization of medial somite identities, with medial expansion of *Pax3* and *Sim* expression domains and loss of *MyoD* expression. This effect was also dose-dependent, as the highest doses of BMP4 completely lateralized the paraxial mesoderm into the lateral plate mesoderm, but medium doses restrained the *Sim*-expressing lateral somite domain (Tonegawa et al., 1997). Conversely, low BMP levels were shown to be crucial for formation of the medial somitic structure and the myotome (Reshef et al., 1998). In zebrafish, overexpression of BMP signaling components increases *Pax3* expression in the dermomyotome and decreases *myoD*-expressing muscle cells (Patterson et al., 2010). In axolotl, insertion of BMP2 beads in the somite results in a dose-dependent disruption of dermomyotome cell and myotome formation (Epperlein et al., 2007). Thus, the BMP signaling pathway is crucial for patterning the dermomyotome across different vertebrate species.

Our analyses demonstrate that BMP signaling patterns the amphioxus somite as well. We observed clear phospho-Smad1/5/8 or phospho-Smad1/5/9 nuclear staining, indicating BMP signaling activity in the lateral portion of the developing amphioxus somite. Furthermore, our inhibitor and exogenous protein experiments show that the lateral domain of amphioxus somite is positively regulated by BMP signaling, similar to that of vertebrates. When BMP signaling was downregulated by Dorsomorphin treatment in amphioxus embryos, ventral outgrowth of the lateral portion of somite was inhibited, and the genes marking the most lateral domain were downregulated (Figure 6); however, the expression of myotome domain genes only exhibited minor changes. Conversely, upregulation of BMP signaling caused the medial expansion of the lateral domain of the somite. Since the shape of the somite have also drastically changed and axial mesoderm of the embryo is deformed or lost, we cannot definitively say that the myotome region is diminished or just merged in the middle. Nevertheless, we show that zBMP4 treated embryo show disorganization of somites along the mediolateral axis. Our results are therefore consistent with studies in axolotl and zebrafish, showing similar functions of BMP signaling in the dermomyotome development (Epperlein et al., 2007; Patterson et al., 2010). Thus, BMP-mediated patterning of the dermomyotome appears to be a conserved feature of the chordate clade.

### *SPARC* Expression Delineates a Lateral Region of the Somite That Contributes to the Formation of Putative Skeletal Tissues in Amphioxus

Previously, the developmental processes of vertebrate endoskeleton and dermal skeleton have been considered to be separate, with inherently disparate histogenesis and ontogenies (reviewed in Hirasawa and Kuratani, 2015). However, a recent review suggests that this categorization is informed simply by the location of the skeletal elements, and not their embryonic development modes (reviewed in Hirasawa and Kuratani, 2015). While this distinction seemingly presents an issue for inferring the homology of different skeletal tissues, the mesothelial “skeletal scaffold” we identified in amphioxus provides another angle to reconcile the supposed incongruence of endoskeleton and dermal skeleton development by uniting both skeletal systems at a single origin in the lateral portion of the somite.

The subepidermal collagenous scaffold in amphioxus may be useful to explain the origin of the dermal skeleton, the first mineralizing skeletal tissue to appear in vertebrates (Wagner and Aspenberg, 2011). The skeletogenic component of the vertebrate dermal skeleton is deposited in the dermis region, which is a derivative of the dermomyotome and lateral plate mesoderm in extant vertebrates (Sire and Huysseune, 2003; Sire et al., 2009). Early dermal bones exhibit a deep osteogenic bone layer, which is capped by superficial odontogenic layers of dentine, enameloid or enamel; the lineages of these two skeletal layers are thought to be patterned independently (Donoghue and Sansom, 2002; Donoghue et al., 2006; Qu et al., 2015; Keating et al., 2018). While the vertebrate cranial dermal skeleton originates from neural crest cells, in the trunk region, both the paraxial mesoderm and neural crest cells contribute to the dermal skeleton. In teleosts, the osteogenic capability of the scales is related to their mesodermal origin (Shimada et al., 2013). In chondrichthyans, CM-DiI lineage tracing revealed the presence of neural crest cells in the cell layer of dermal denticles (Gillis et al., 2017). The authors of that study therefore proposed an osteo-odonto dichotomy to demarcate the mesoderm and trunk neural crest cell contributions in the dermal skeleton (Gillis et al., 2017). The mesoderm origin of the amphioxus dermal skeleton-like structure thus suggests that the mesoderm-based portion of the dermal skeleton (the deeper osteogenic layers) may be an ancestral character of the chordate clade. Because amphioxus does not have neural crest cells, this mesodermal origin also implies that the ability of neural crest cells to form exoskeletal tissues may have resulted from co-option of the skeletogenic developmental program from the mesoderm in the vertebrate lineage, consistent with a previously proposed hypothesis (Jandzik et al., 2015).

**FIGURE 8:**
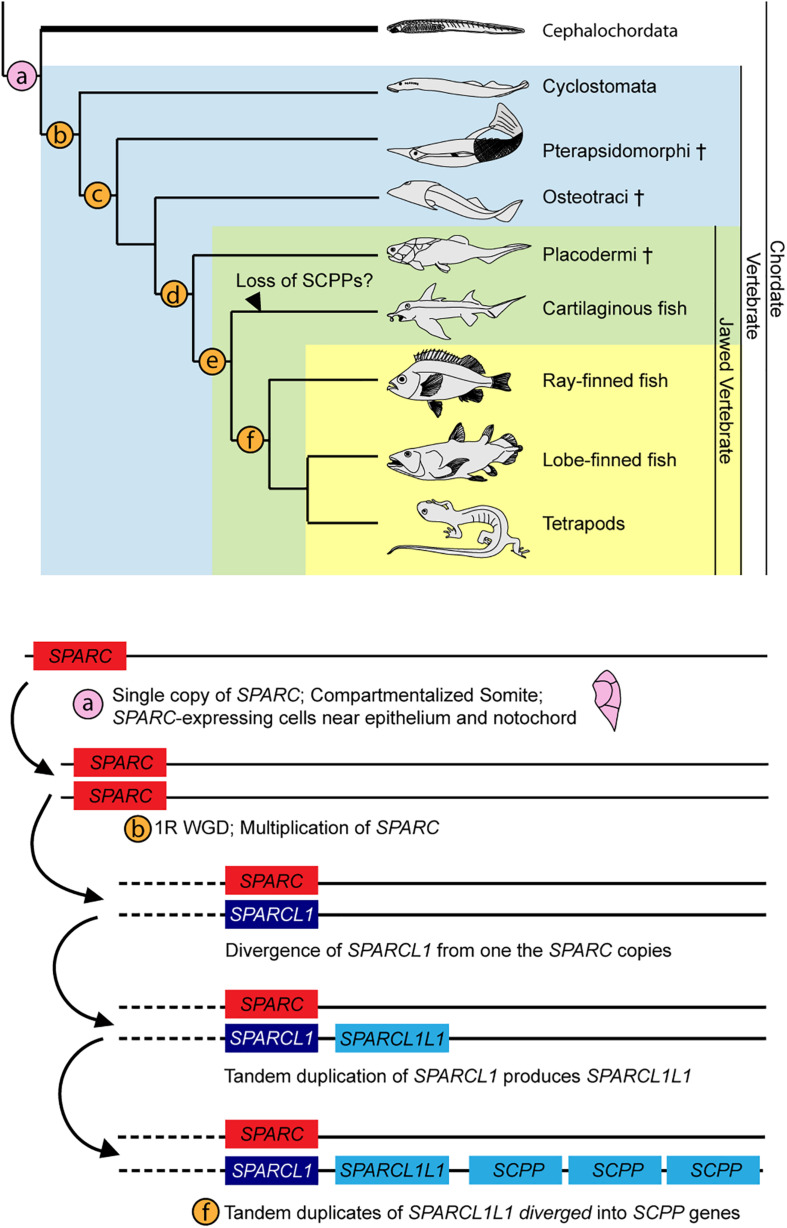
Summary of SPARC family expansion throughout chordate phylum. Cross indicates extinct taxa. Phylogenetic tree is adapted from Kawasaki and Weiss (2006). Expansion of SPARC family is adapted from Kawasaki and Weiss (2006), with updates from findings in Kawasaki (2018). **(a)** Somite compartmentalization is present in the common ancestor of all chordates. Mesoderm-derived rudimentary collagenous skeleton ensheaths the outer layer of the somite and the notochord. Cartilaginous tissues are found in the oral cirri (cellular) and pharyngeal region (acellular) (Rychel et al., 2005; Jandzik et al., 2015). **(b)** 1R of whole genome duplication. *Sparca* and *Sparcb* in lamprey co-orthologs of Sparc (Bertrand et al., 2013; Kawasaki, 2018; Simakov et al., 2020). **(c)** All four types of skeletal tissue (cartilage, bone, dentine, enameloid) found (Keating et al., 2018). **(d)** 2R of whole genome duplication (Simakov et al., 2020). **(e)** *SPARCL1L1* (*SPARCL2* in Enault et al., 2018) in chondrichtyhans is likely originated from an asymmetric duplication of *Sparcb* (Kawasaki et al., 2017; Kawasaki, 2018). **(f)** *SPARCL1L1* tandem duplicates, subsequent copies diverged into the SCPP gene family (Kawasaki et al., 2017; Kawasaki, 2018).

SPARC is a major ECM (Extracellular matrix) component in bone and dentine (Kawasaki and Weiss, 2008), and the protein contains three domains: a 5′ N-terminus that binds Ca^2+^ with low affinity but high capacity, a middle follistatin-like domain, and a C-terminus containing two EF fingers with high calcium affinity (Bradshaw, 2009; Rosset and Bradshaw, 2016). The C-terminal domain of SPARC also binds to collagen during ECM formation (Hohenester et al., 1997). In vertebrates, diversification and hypermineralization of skeletal elements have been attributed to the expansion of the *SPARC* family genes during two rounds of vertebrate whole genome duplications (Kawasaki et al., 2004; Kawasaki and Weiss, 2006; Putnam et al., 2008; Simakov et al., 2020). After the first *SPARC* duplication, one of the copies diverged to become *SPARCL1* (Kawasaki et al., 2004, 2017), which then underwent tandem duplication, and one of the diverged duplicates, called *SPARCL1L1*, further duplicated to generate various *SCPP* genes (Kawasaki, 2018). SCPP proteins are heavily involved in producing hypermineralized odontogenic structures (e.g., teeth, dermal denticles and bones) by attracting different mineralizing repertoires to the existing ECM framework (Donoghue et al., 2006; Kawasaki, 2009). The presence of *SPARC* expression in the lateral portion of the amphioxus somite and mesothelial layer provides additional support for the proposed enamel-enameloid-dentine-bone continuum (Donoghue et al., 2006; Kawasaki and Weiss, 2008). We propose that the amphioxus *SPARC*-expressing rudimentary skeletal scaffold may represent an ancestral condition in chordates, with the skeletal scaffold acting pivotally during subsequent elaborations of vertebrate hypermineralization (Figure 8).

Aside from its role in mineralization, *SPARC* also plays an important role in maintaining the integrity of extracellular matrix in bilaterians (Bradshaw, 2009; Chioran et al., 2017). In *SPARC*-null mouse, the collagen fibrils in the dermis become shorter and less tensile, resulting in abnormal dermis development (Bradshaw et al., 2003). In Drosophila, *SPARC*-expressing hemocytes maintain the integrity of the basal laminae; *SPARC*-mutant do not have Collagen IV in the basal laminae and laminin network is disrupted (Martinek et al., 2008). In *C. elegens*, *SPARC* is expressed in the body wall, sex muscle and gonad whereupon downregulation results in larval lethality, transparency in surviving progenies, and infertility or reduced fecundity (Fitzgerald and Schwarzbauer, 1998). Since the amphioxus dermal skeletal scaffold does not mineralize, we postulate that *SPARC* might play an important role in maintaining the integrity of the closely associated collagenous scaffold. This also implies that the function of *SPARC* in maintaining the ECM is a conserved feature of all bilaterians. Interestingly, *SPARC* is also found in mineralized non-chordate invertebrates such as the sea urchin, which is thought to produce dissimilar mineralizing repertoire to the vertebrate (Livingston et al., 2006). Further investigations of the role of *SPARC* in mineralizing non-chordates may also reveal various deep homologies between the mineralized structures.

In a broader phylogenetic context, our results in amphioxus may provide additional insights into the relationships between various skeletal elements in vertebrates and skeleton-like tissues in invertebrates, such as pharyngeal cartilage in hemichordates, cartilage-like tissues in polychetes, cartilage tissues in mollusks and arthropods, and even the collagenous ECM structure secreted by the annelid axochord (Cole and Hall, 2004; Rychel et al., 2006; Lauri et al., 2014). It will be important to further examine whether the cells that produce either dense collagen-based ECM or skeletal elements across diverse metazoan phyla are all (i) derived from mesoderm progenitors and (ii) employ homologous gene regulatory networks for their specification and patterning. Answering these questions will help us further understand the evolutionary origin of skeletogenic cells within metazoan animals.

## Materials and Methods

### Animals, Embryos, and Drug Treatments

Florida amphioxus (*Branchiostoma floridae*) adults were collected in Tampa Bay, Florida, United States, during the summer breeding season. Gametes were obtained by electrical stimulation and spontaneous spawning. Fertilization and subsequent culturing of the embryos were carried out as previously described (Yu and Holland, 2009). Amphioxus embryos were staged according to Carvalho et al. (2021). BMP signaling was manipulated by treating embryos with recombinant zebrafish BMP-4 protein (zBMP4, R&D Systems) at 250 ng/ml, as previously described, or by treating with the small-molecular inhibitor, Dorsomorphin (Sigma) (Yu et al., 2007). Dorsomorphin was dissolved in DMSO to prepare a 10 mM stock solution, and experimental embryos were treated at 16 μM from the G4 stage, as previously described (Lu et al., 2012). Control embryos were treated with an equal amount of DMSO or 0.1% BSA in filtered seawater. Experiments were carried out as described in Yong et al. (2019).

### Amphioxus *in situ* Hybridization and Immunostaining

Polymerase chain reaction (PCR) was performed to amplify full length cDNAs of *B. floridae* genes from an EST library as previously described (Yu et al., 2008), and the PCR products were used as templates for synthesizing DIG-labeled antisense riboprobes (Wu et al., 2011). Information regarding the EST clones used in this study is provided in Supplementary Table 1. Embryo fixation, *in situ* hybridization, and immunostaining were done as previously described, with minor modifications (Beaster-Jones et al., 2008; Lu et al., 2012; Li et al., 2014). For synthesizing DIG-labeled antisense riboprobe for *Col4a1/3/5* gene (cDNA clone: bflv046m21), we used pDONR222-SP6-Forward primer and a gene-specific primer with T7 promoter site (5′-TAATAC GACTCACTATAGGGAGACAAGCCCATGTCACCTTTCT-3′) to amplify a 1kb fragment as template; riboprobe was synthesized using T7 RNA polymerase. *B. floridae Tbx1/10* cDNA, which could not be identified in the EST database, was amplified by PCR using a cDNA library (a kind gift from Dr. Jim Langeland) constructed in the pBluescript vector. PCR was performed using the KAPA Long Range PCR Kit and primers pBluscript-*Not*I (5′-CTTGCGGCCGCTCACTATAGGGCCAA TTGGGTACC-3′) and *Tbx1/10*-reverse1, which contains a *Bam*HI cutting site (5′-CGGGATCCCGTAACATGTGAAGA TACGGAAATGG-3′). To generate the template for the antisense riboprobe, primers pBluescript-*Not*I and Tbx1/10_T7_reverse, which has a T7 binding site (5′-TAATACGACTCACTA TAGGGTAACATGTGAAGATACGGAAATGG-3′), were used for PCR. The purified PCR products were used to synthesize DIG-labeled antisense riboprobes using T7 RNA polymerase. For all *in situ* hybridization experiments: pre-hybridization was performed for 4 h at 60°C; hybridization was done at 65°C; the RNase step was conducted for 30 min at 37°C. For *Pax3/7* in particular, pre-hybridization was done at 60°C overnight, and hybridization was done at 60°C overnight; the RNase step was 20 min. For double-color *in situ* hybridization of amphioxus embryos, *SPARC* and *mActin* cDNA fragments were amplified by PCR to synthesize fluorescein-labeled and DIG-labeled antisense riboprobes. After hybridization and washes with an SSC gradient, embryos were incubated in blocking solution (2 mg/ml BSA in PBST with 10% volume of sheep serum) for 1 h. Afterward, embryos were incubated in antibody solution containing alkaline phosphatase (AP)-conjugated anti-DIG antibody (Roche) at 4°C overnight; Fast Red tablets (Sigma) were used for the first color (red) development. Dissolved tablets were filtered through a 0.22-μm filter to prevent precipitation. Red color development for *mActin* was followed by treatment with antibody killer solution (0.1 M glycine, pH 2.2 in PBST) for 5 min, and four washes in PBST for 5 min each. After blocking for 1 h and incubating in anti-fluorescein-AP antibody solution at 4°C overnight, *SPARC* expression was visualized with NBT/BCIP (second color: dark purple) development. Some darkly stained embryos were embedded in Tissue-Tek Optimal Cutting Temperature (O.C.T.) compound (Sakura, Japan), and cryosectioned (8–10 μm thickness) on a cryostat (CM3050s, Leica). Images of Cryosection were captured with a Zeiss AxioCam HRc CCD camera. The same DIC images were also imaged with Hoechst (Invitrogen, 1 μg/mL in PBST) on the same level. Superimposed Hoechst-DIC images were done with FIJI. Superimposed condition was tailored for each individual section to maximize the display of the nuclei without altering the original DIC images. Changes are uniformly applied for the same sample.

To detect *SPARC* expression in amphioxus juveniles, fixed samples (∼0.5 cm long) were embedded in O.C.T. compound and cryosectioned (16 μm thickness) on a cryostat, thaw-mounted on glass slides (MAS-GP type A coated glass slide, Matsunami), and stored at −20°C. *In situ* hybridization of cryosection samples was performed as for whole-mount samples, but with the following modification: cryosections were thawed, dried at 37°C for 1 h, and washed in PBST three times; proteinase K treatment was omitted and the samples were rinsed in 0.1 M triethanolamine before proceeding with the acetic anhydride treatment. The rest of the procedure was the same as the described *in situ* hybridization method. Images of cryosections were captured with a Zeiss AxioCam MRc CCD camera mounted on a Zeiss Imager A2 microscope.

Double fluorescent *in situ* hybridization and double fluorescent staining of *Hu/elav* RNA and pSmad1/5/8 protein (primary antibody: pSmad1/5/8, Cell Signaling #9511, pSmad1/5/9 Cell Signaling #13820, 1:200) were performed as previously described (Wu et al., 2011; Lu et al., 2012; Yong et al., 2019). Signals were detected using secondary antibodies (Alexa flour 594 or 555 goat-anti-rabbit antibody for pSmad1/5/8 or pSmad1/5/9, 1:400). DAPI or Hoechst (Invitrogen, 1 μg/ml in PBST) was used for nuclear staining. Fluorescence images were acquired using a Leica TCS-SP5 confocal microscope or a Zeiss LSM880 Inverted confocal microscope.

### Transmission Electron Microscopy (TEM)

Amphioxus young juveniles (21-days-old) were fixed in 0.2 M phosphate buffer (pH 7.3) with 2.5% glutaraldehyde at 4°C overnight. The samples were then post-fixed in 4% osmic acid for 4 h at 4°C, washed in 0.2 M Phosphate buffer (pH 7.3) three times (1 h each), and dehydrated through a graded ethanol series (30, 50, 70, 90, and 100%, 30 min each) at room temperature before being washed three times with 100% acetone. The samples were embedded first in 1:2 Spurr Low-Viscosity Embedding media (Sigma-Aldrich), and then in 1:1 and then 2:1 proportions of the same embedding media (2 h/step). Subsequently, the samples were left in pure media overnight and then cured for 8 h in a 70°C oven. The hardened blocks were cut into ultrathin sections using a Leica EM UC7 ultramicrotome, and the sections were contrasted with uranyl acetate and lead citrate. The sections were examined and photographed under a Hitachi 7000 (HITACHI, Tokyo, Japan) transmission electron microscope (TEM) equipped with a CCD camera. Amphioxus juvenile animals (1 cm length) were fixed in 2% paraformaldehyde and 2.5% glutaraldehyde in 0.1 M cacodylate buffer overnight. Animals then were rinsed three times with 0.1 M cacodylate buffer (15 min/rinse), followed by post-fixation in 1% osmium tetroxide for 1–2 h. After three more washes in 0.1 M cacodylate buffer, the specimens were dehydrated using graded ethanol series, and then incubated three times (10 min each) with fresh propylene oxide (PO). Next, the specimens were transferred to PO/resin (Embed 812/Araldite) as a 1:1 mixture and left in a vacuum overnight, before being embedded in resin for 8 h in a vacuum. The embedded specimens were hardened in a 62°C oven for 48–72 h. The blocks were trimmed, and 80-nm ultrathin sections were cut using a diamond knife. The sections were examined and photographed under a Hitachi HT7700 TEM equipped with a CCD camera.

## Data Availability Statement

The original contributions presented in the study are included in the article/Supplementary Material, further inquiries can be directed to the corresponding author/s.

## Author Contributions

J-KY conceived and designed the study. LWY, T-ML, K-LL, and J-KY performed embryonic experiments, performed image acquisition, and data analysis. LWY, T-ML, and K-LL performed *in situ* hybridization and immunostaining. C-HT and R-JC performed TEM. LWY, T-ML, and J-KY assembled the figures and wrote the manuscript draft. All authors have read and approved the final manuscript.

## Conflict of Interest

The authors declare that the research was conducted in the absence of any commercial or financial relationships that could be construed as a potential conflict of interest.
