# Supplementary material for: Somite Compartments in Amphioxus and Its Implications on the Evolution of the Vertebrate Skeletal Tissues
Source: Front Cell Dev Biol. 2021 May 10;9:607057. doi: 10.3389/fcell.2021.607057 (PMC8141804; doi:10.3389/fcell.2021.607057)
Supplement: Supplementary file 1 [file Data_Sheet_1.PDF]

Supplementary Data: includes material and methods section, 2 tables, 10 figures and 1 reference list

Supplementary Materials and Methods:

Phylogenetic analysis of amphioxus Collagen4 genes:
NC1 domain of the Collagen4 protein were deduced using SMART (Letunic et al., 2014). NC1 domain alignment are done using the MUSCLE algorithm on MEGA X (Edgar, 2004; Stecher et al., 2020). The following protein sequences were used for the analysis: HsCol2a1: NP_001835.3; HsCol4A1: NP_001836.2; HsCol4a2: NP_001837.2; HsCol4a3: NP_000082.2; HsCol4a4: NP_000083.3, HsCol4a5: NP_000486.1, HsCol4a6: NP_001838.2, MmCol4a1: NP_034061.2, MmCol4a2: NP_034062.3, MmCol4a3: NP_031760.2, MmCol4a4: NP_031761.1, MmCol4a5: NP_001156627.1, MmCol4a6: NP_444415.2, GgCol4a1: NP_001155871.1; GgCol4a2: NP_001155862.1, DsCol4a1: NP_723044.1, RnCol4a1: NP_001128481.1; BtCol4a1: NP_001159983.2; CfCol4a5: NP_001002979.1; GgCol4a3: AAY43819.1; RnCol4a3: NP_001129231.1; BfCollagen4a1/3/5: MW574135; BfCollagen4a2/4/6: MW574134
Hs: *Homo sapiens*, Mm: *Mus Musculus*, Gg: *Gallus gallus*, Dm: *Drosophila melanogaster*, Rn: *Rattus norvegicus*, Bt: *Bos taurus*, Cf: *Canis familaris*, Bf: *Branchiostoma floridae*

Amino acid substitution was analyzed using MEGA X (Stecher et al., 2020). Maximum-likelihood phylogenetic tree were inferred with RAxML-HPC BlackBox (8.2.12) via the CIPRES Science Gateway V.3.3, using the LG model (Stamatakis, 2014). Bootstrap inferences were executed 1000 times. The results are displayed with FigTree v1.4.4. The ML tree is rooted at midpoint. Node indicates bootstrap value.

Fluorescent Wholemount in situ hybridization and pSMAD1/5/9 staining:
Fluorescent wholemount in situ hybridization and pSMAD1/5/9 are performed as mentioned in the materials and methods section. Images of orthogonal sections are obtained with Imaris x64 9.6.0. Brightness levels are uniformly applied across all images and between treatments to ensure comparable conditions.

Table S1: List of EST library clones used to generate *In Situ* hybridization probes

Gene	EST clone	Citations
SPARC	bfeg032g12	(Bertrand et al., 2013)
mActin	bfne159i15	(Holland et al., 1995)
Zic	bfne006l12	(Gostling and Shimeld, 2003)
FoxF	bfne015j03	(Onimaru et al., 2011)
Twist	bfne115j15	(Meulemans and Bronner-Fraser, 2007)
Bapx	bflv046h23	(Meulemans and Bronner-Fraser, 2007)
Runx	bfne142d14	(Meulemans and Bronner-Fraser, 2007)
Hand	bfga046k20	(Onimaru et al., 2011)
MRF2	bfne071g19	(Schubert et al., 2003)
MRF1	bfne030a14	(Schubert et al., 2001)
FoxC	bfne075l20	(Aldea et al., 2015)
Pax1/9	bflv043h07	(Kozmik et al., 2007)
SoxE	bfne111n24	(Meulemans and Bronner-Fraser, 2007)
Ets	bfad008i08	(Meulemans and Bronner-Fraser, 2007)
Pax3/7	bfne104i03	(Holland et al., 1999)
Hu/Elav	bfne129i05	(Lu et al., 2012)
Col4a1/3/5	bflv046m21	*This study*
Col4a2/4/6	bfne082c03	*This study*

Table S2: **Summary of dermomyotome homologs in vertebrates and their roles in dermomyotome patterning and development**. An argument for a dermomyotome in teleosts is reviewed in (Devoto et al., 2006). This list does not provide exhaustive information about the listed genes. DML, dorsomedial lip; VML ventromedial.

**Amphioxus Homolog**		**Vertebrate homolog**	**Expression pattern in Dermomyotome / Derivatives**	**Function/Interaction**
Pax3/7 (Holland et al., 1999)	Chick	Pax3	DML & VML of dermomyotome (Williams and Ordahl, 1994;Ben-Yair and Kalcheim, 2005)	Ectopic *Pax3* leads to ectopic expression of *MyoD* and *Myf5* in neural tube and lateral plate mesoderm (Maroto et al., 1997)
		Pax7	Mainly central Dermomyotome, satellite cells (Ben-Yair and Kalcheim, 2005)	
	Mouse	Pax3	Dermomyotome (Horst et al., 2006)	Pax3 mutants lose limb musculature but trunk musculature is not affected (Goulding et al., 1994)
		Pax7	Dermomyotome (Jostes et al., 1990), Satelite Cells (Horst et al., 2006)	Pax7 is functionally redundant in maintaining dermomyotome and primary myotome but does not perform the role of Pax3 in limb myogenesis. (Relaix et al., 2004)
	Zebrafish	Pax3	Anterior Border cells and lateral cells (Hammond et al., 2007)	
		Pax7	Anterior Border Cell and lateral cells (Hammond et al., 2007; Stellabotte et al., 2007)	
	Frogs	Pax3	Dermomyotome (Grimaldi et al., 2004)	
		Pax7	Dermomyotome (Grimaldi et al., 2004)	
	Lamprey	Pax3/7	Dermomyotome (Kusakabe and Kuratani, 2005)	
	Hagfish	Pax3/7	Dermomyotome (Ota et al., 2011)	

Zic (Gostling and Shimeld, 2003)	Chick	Zic1	DML of dermomyotome (Sun Rhodes and Merzdorf, 2006)	
		Zic2	DML of dermomyotome and periotic mesoderm (McMahon and Merzdorf, 2010)	
		Zic3	DML of dermomyotome (McMahon and Merzdorf, 2010)	
	Mouse	Zic1	Dorsomedial portion of somite (Nagai et al., 1997; Pan et al., 2011)	*Zic1* coordinates with *Pax3 and Gli3* to activate promoter of *Myf5* in functional assay (Himeda et al., 2013)<br><br>*Zic1* induces Myf5 expression in functional assay and presomitic mesoderm explants (Pan et al., 2011)
		Zic2	Dorsomedial portion of somite (Nagai et al., 1997; Pan et al., 2011)	Antibody staining reveals co-localization with *Pax3* and Myf5 in DML of dermomyotome (Pan et al., 2011)<br><br>Zic1 and Zic2 facilitate Gli-dependent activation of Myf5 epaxial somite specific enhancer in functional assay (Pan et al., 2011)
		Zic3	Dorsomedial portion of somite (Nagai et al., 1997; Pan et al., 2011)	
	Zebrafish	Zic1	Dorsomedial portion of somite (Rohr et al., 1999)	
Twist (Meulemans and Bronner-Fraser, 2007)	Chick	cDermo-1 (Twist2)	Dermis progenitor, dermomyotome (Scaal et al., 2001; Hornik et al., 2005)	cDermo-1 Ectopic expression of *Dermo* causes formation of dense dermis in pterylae (Hornik et al., 2005)

	Mouse	Twist	Dermomyotome (Füchtbauer, 1995), Dermis progenitor	Appears to suppress myogenesis in somite (Füchtbauer, 1995)
	Mouse	Dermo-1	Dermomyotome, Dermis progenitor (Li et al., 1995)	
	Trout	*Dermo*1	Dermomyotome (Dumont et al., 2008)	

Figure S1: Multi-colored fluorescent *in situ* hybridization of sections from N3 stage embryos. (A-C) *mActin* is expressed in the medial region **(A)**, while *SPARC* **(B)** is expressed in the lateral part of the somite. The expression domains are largely non-overlapping **(C)**. **(D)** *MRF2* expression overlaps with the *mActin* expression domain. **(E)** *Pax3/7* is expressed in the lateral part of the somite, forming a separate domain from the myotome marker *mActin*. **(F)**. Both *Zic* and *Pax3/7* have overlapping domains in the lateral part of the somite. **(G-I)** The lateral part of the somite is further partitioned into three components. **(G)** *Twist* is expressed in both the ventrolateral and centrolateral parts of the somite but not the dorsolateral part, and its expression domain does not overlap with that of *mActin*. **(H)** *FoxF* is only expressed in the ventro-lateral portion of the somite. **(I)** *Tbx1/10* is expressed in the ventrolateral and centrolateral parts of the somite in the 2nd and 3rd somite. It is also expressed in the myotome part of the somite, overlapping with the *mActin* expression domain.

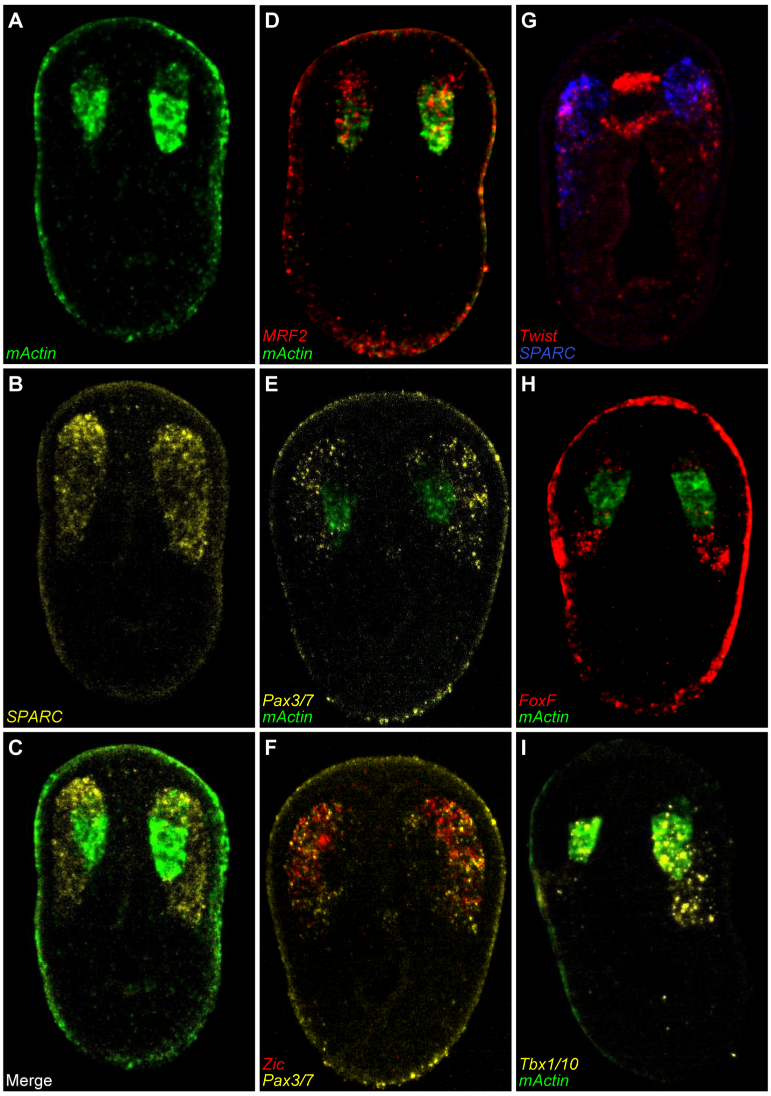

Figure S2: Expression of *Tbx1/10* upon perturbation of BMP Signaling pathway. Magenta line marks the second somite, where section A', B', C' are obtained. White brackets mark the 6th to the 8th somite where the sections A", B", C" are obtained. **(A-A'')** In the anterior region of the embryo, *Tbx1/10* is expressed in the myotome-like region as well as the centrolateral and ventrolateral regions (A'). In the posterior part, it is expressed in the more ventral portions of the myotome-like region (A''). **(B-B'')** Inhibition of BMP Signaling with inhibitor Dorsomorphin causes a loss of the lateral expression domain (B and B', compared to A and A'), while in the more posterior part of the embryo, myotome domain expression remains (B''). **(C-C")** Overexpression of BMP with recombinant zBMP4 protein causes the expansion of the lateral expression domain, while in the posterior part of the embryo, myotome domain is merged in the midline (C'').

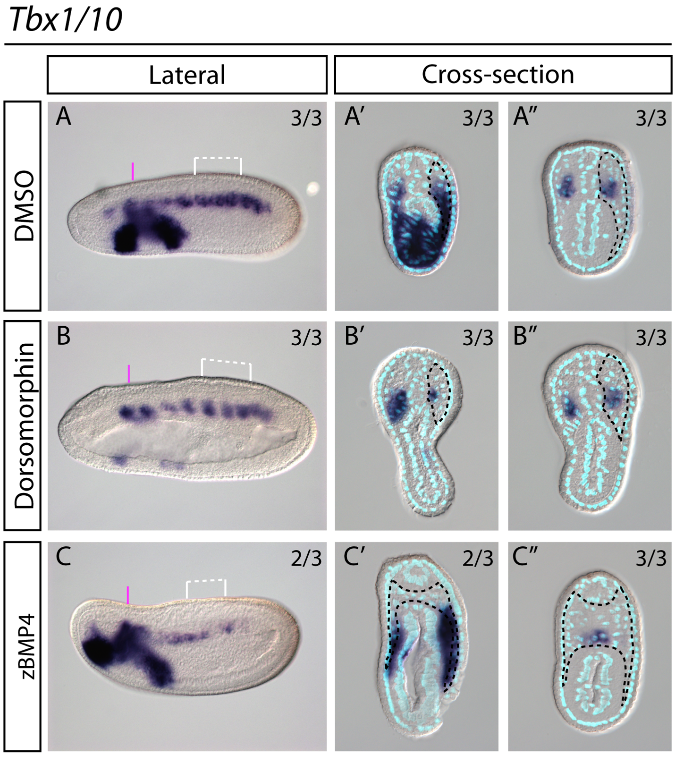

Figure S3: Cross section of Dorsomorphin treatment and control embryo from anterior tip to posterior tip **(A-B)** Hoechst staining of sections of DMSO Control and Dorsomorphin treatment embryo. Yellow lines mark the ventral most boundary of somite ventral expansion. Section sequence are indicated in top right corner.

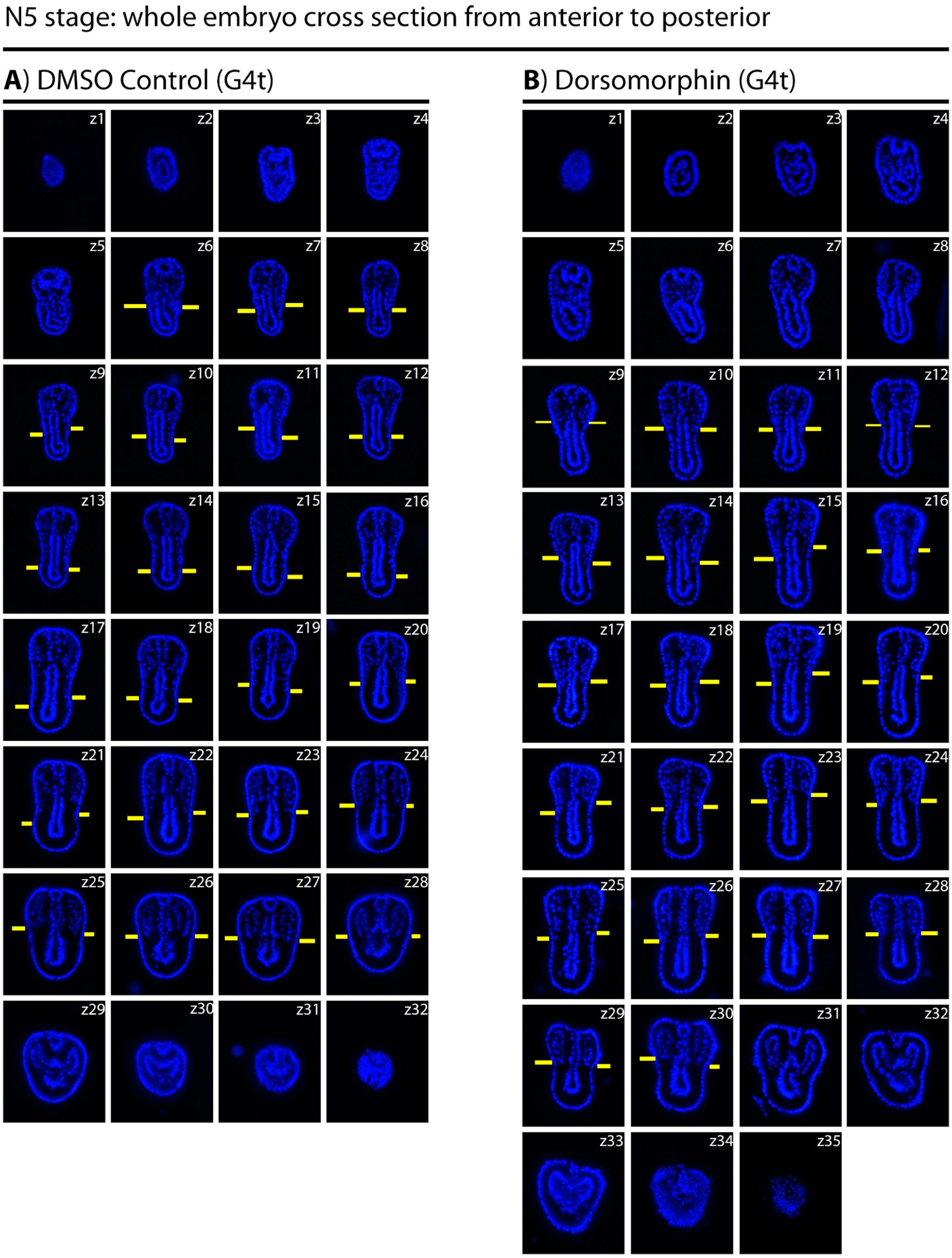

Figure S4: Whole-mount embryos for BMP signaling treatment experiment: *SPARC* + *mActin*. Cross section is an alternate display of images from main figure 5**(B-D)**. White brackets indicate the region where the cross sections are picked from, corresponding to the 4th til the 6th pair of somite.

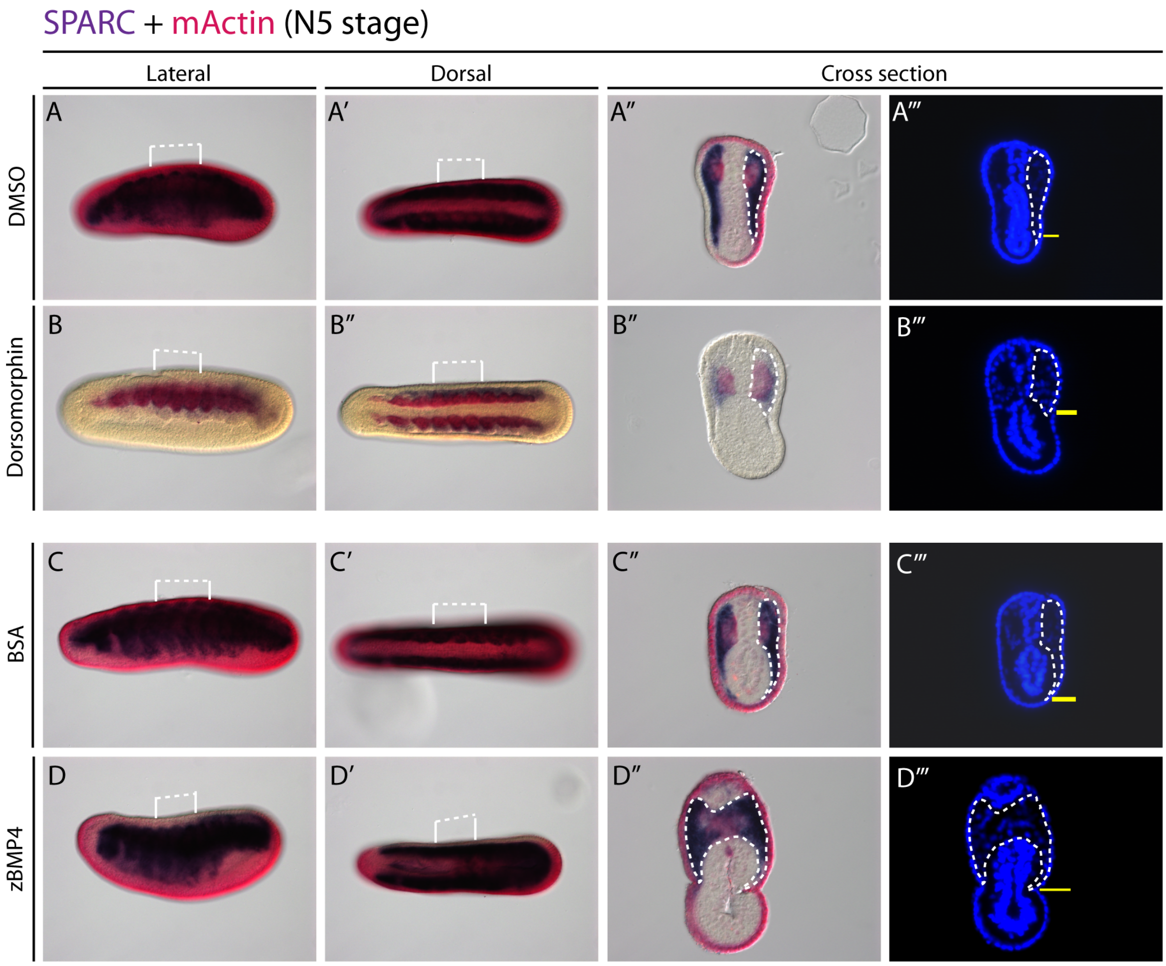

Figure S5: Wholemount embryos for BMP signaling treatment experiment: *Zic, Twist*. Different alphabet on the same treatment represent one embryo.

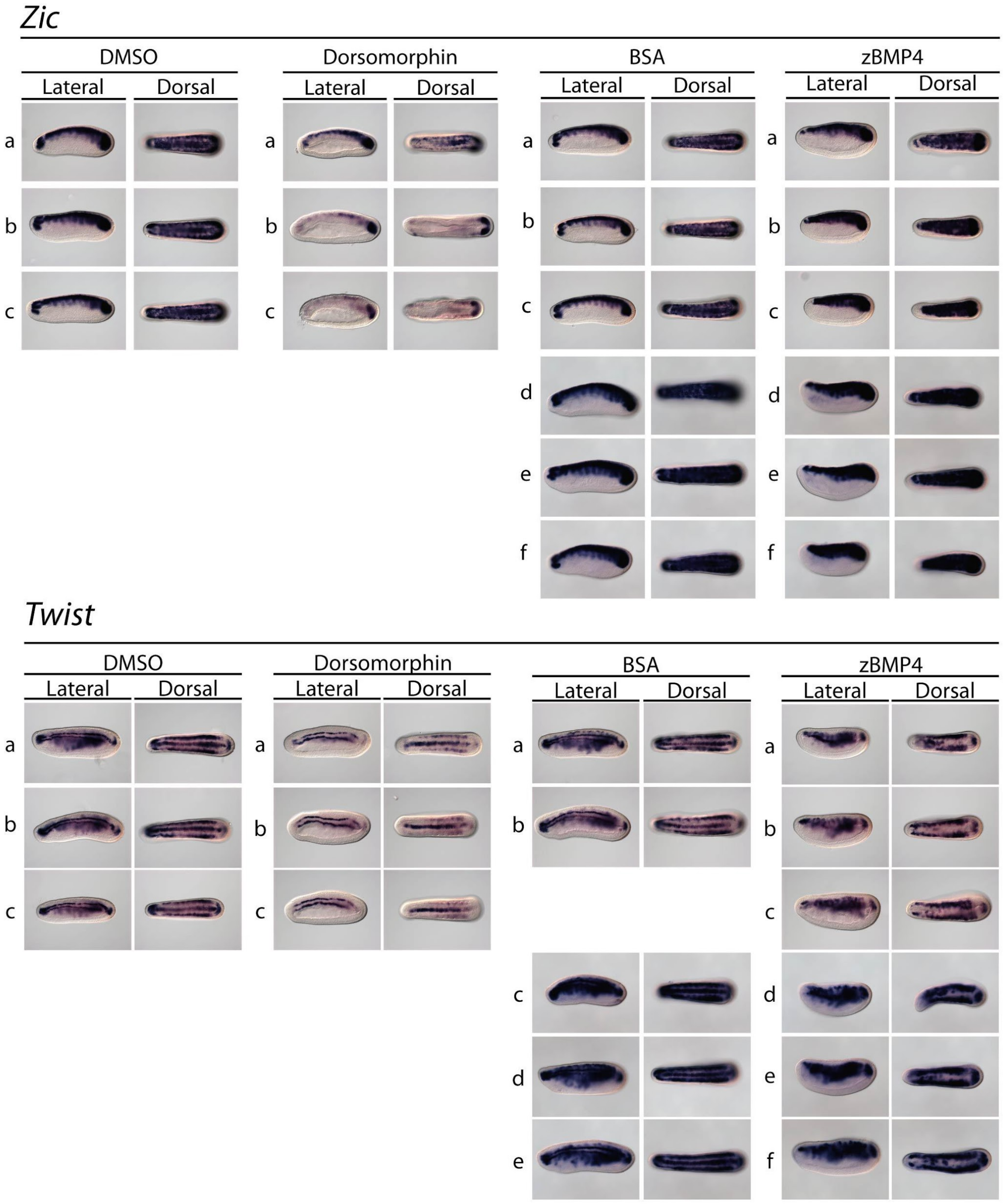

Figure S6: Wholemount embryos for BMP signaling treatment experiment: *Pax3/7*. Different alphabet on the same treatment represent one embryo.

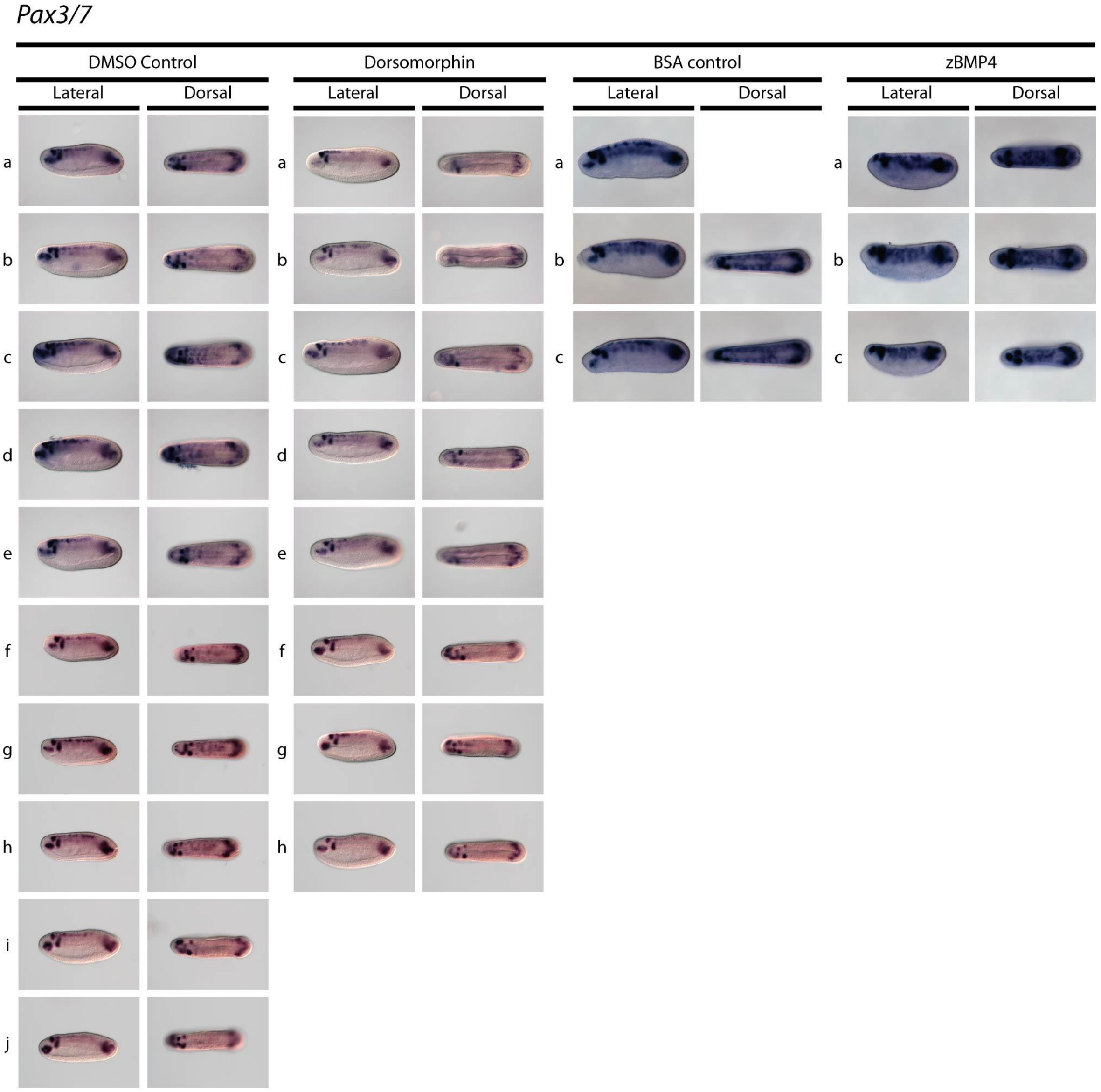

Figure S7: Wholemount embryos for BMP signaling treatment experiment: *Hand, FoxF*. Different alphabet on the same treatment represent one embryo.

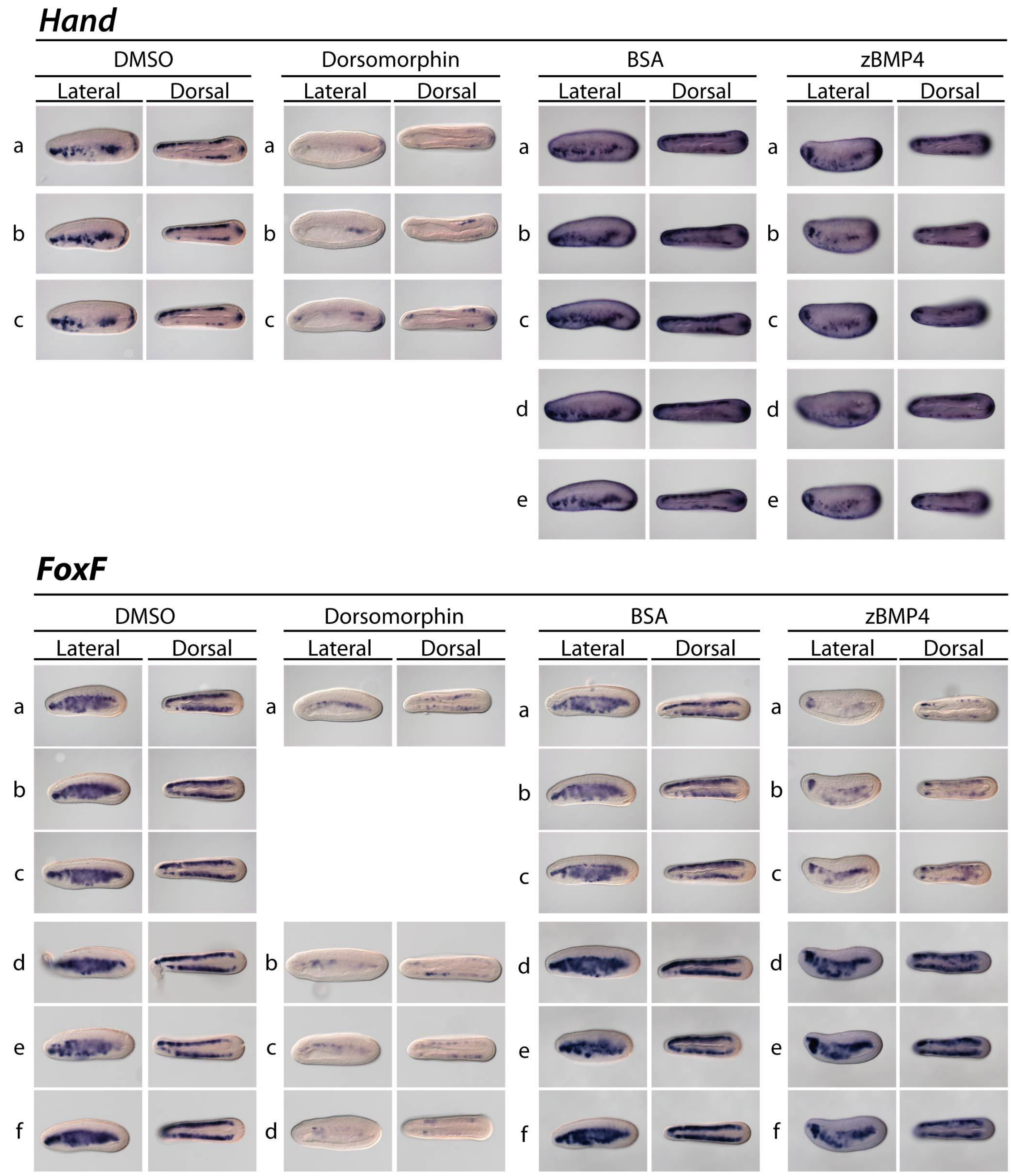

Figure S8: Wholemount embryos for BMP signaling treatment experiment: *MRF1, Tbx1/10, FoxC*. Different alphabet on the same treatment represent one embryo.

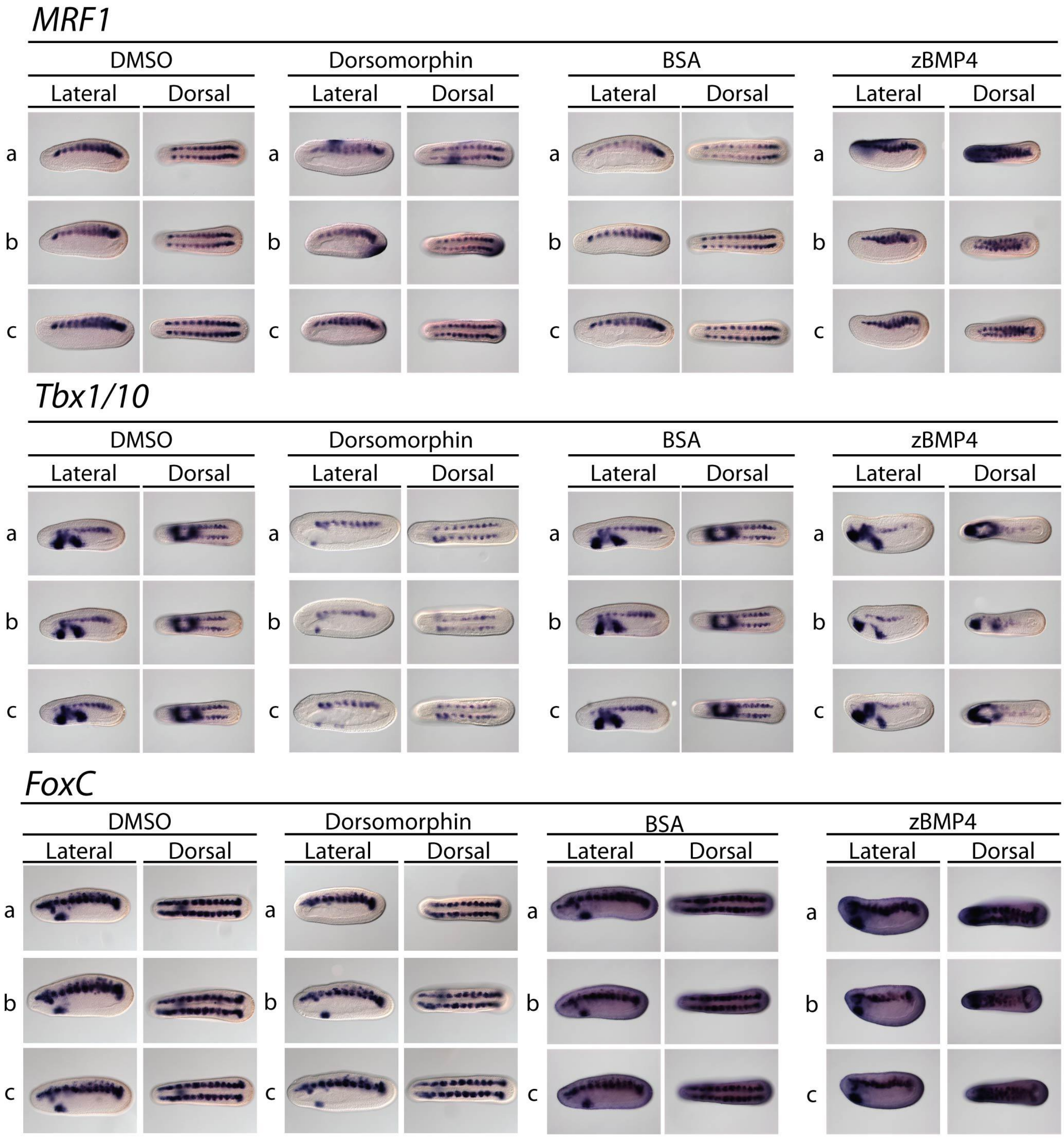

Figure S9: Maximum Likelihood phylogenetic tree of amphioxus Collagen4. Collagen4a1/3/5 is assigned to the EST clone bfne082c03. Collagen4a2/4/6 is assigned to the EST clone bflv046m2. Hs: *Homo sapiens*, Mm: *Mus Musculus*, Gg: *Gallus gallus*, Dm: *Drosophila melanogaster*, Rn: *Rattus norvegicus*, Bt: *Bos taurus*, Cf: *Canis familaris*; Numbers in node represent bootstrap value. Tree is rooted in the midpoint.

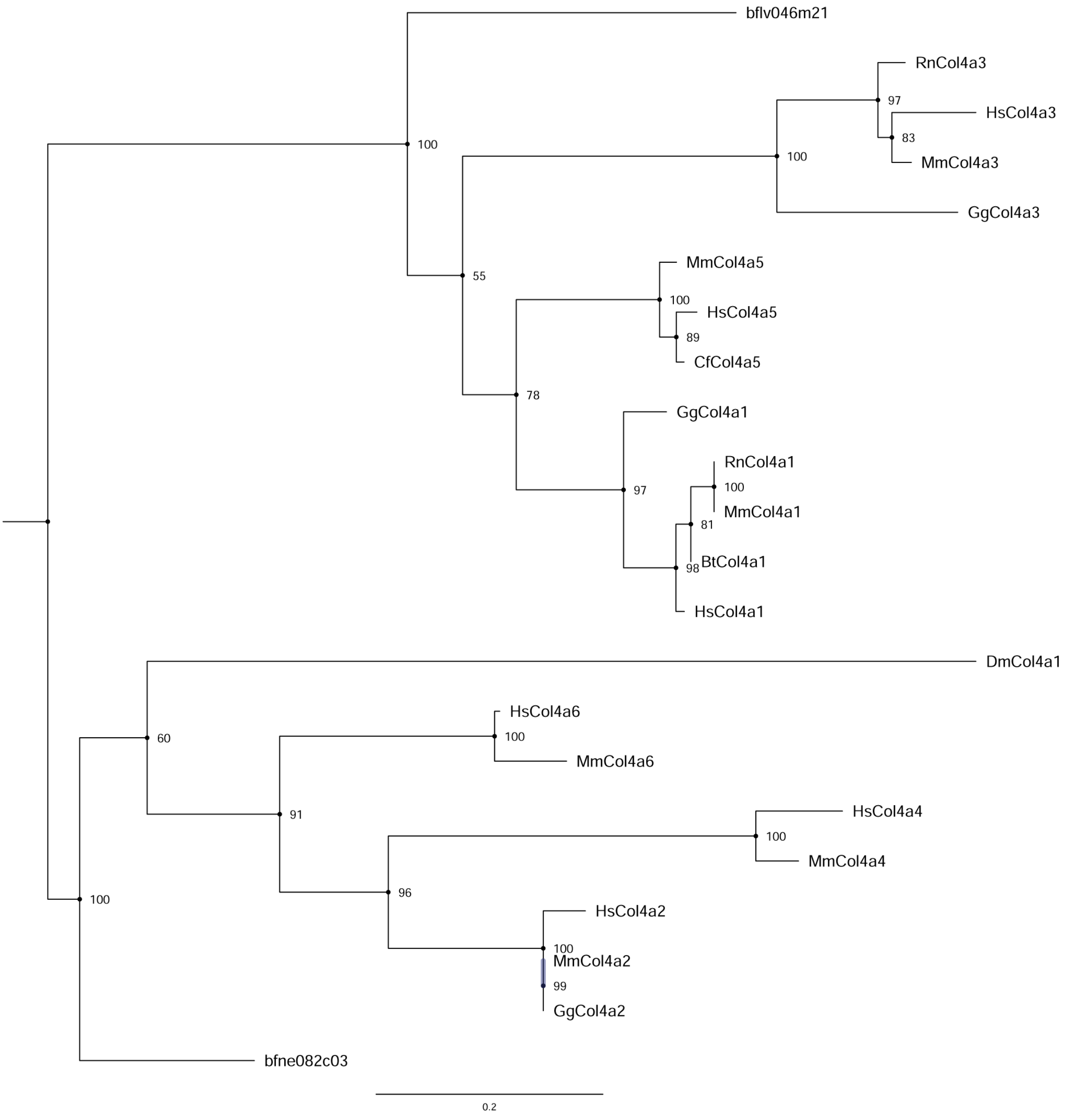

Figure S10: Orthogonal view of *FoxF* transcripts + pSMAD1/5/9 co-localization in BMP signaling perturbed embryos. Cyan arrow marked the widest portion of the embryo and also the section where the somite exhibit the ventral-most expansion. White arrow marks the section taken from the 7th somite level. **(A-C")** *FoxF* expression domain (a lateral somite marker) shows that the ventro-lateral domain of the somite exhibits ventral expansion. **(D-E")** When treated with Dorsomorphin, this FoxF expression level is diminished and the somite does not expand ventrally; **(F-F")** concurrently, pSMAD1/5/9 signal is also lower compared to the control. **(G-I")** In zBMP4 treated embryos, the ventral expansion of this *FoxF* expressing domain is affected and remains concentrated in the medial portion of the embryo. pSMAD1/5/9 staining is also more concentrated in the medial portion of the embryo.

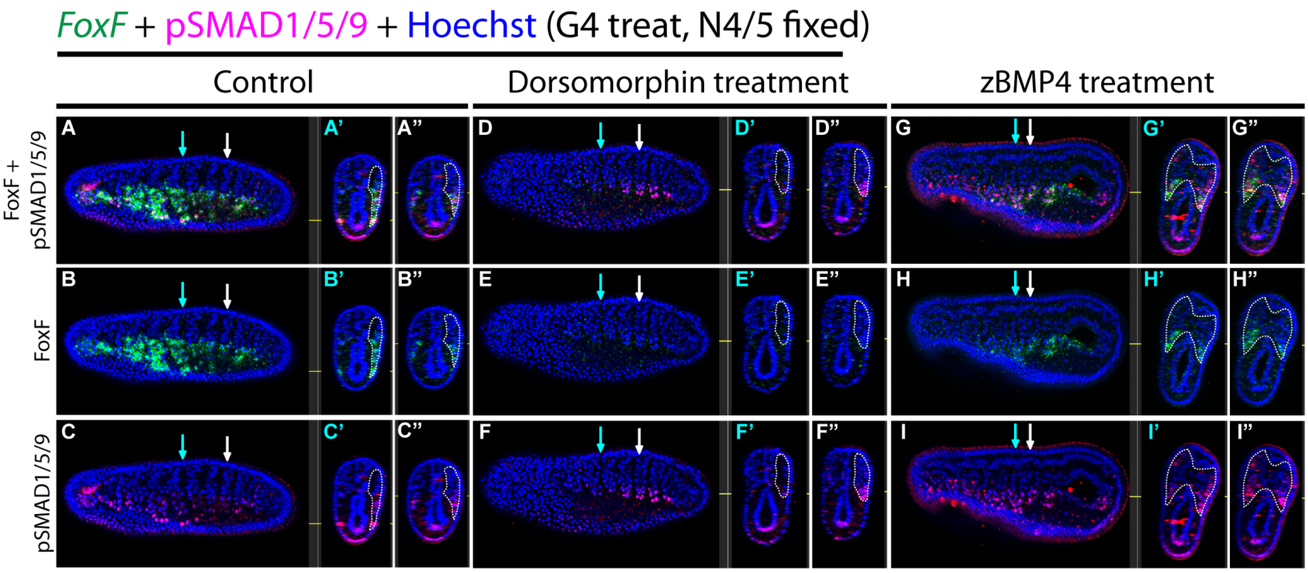

Supplementary Reference

Aldea, D., Leon, A., Bertrand, S., and Escriva, H. (2015). Expression of Fox genes in the cephalochordate Branchiostoma lanceolatum. *Frontiers in Ecology and Evolution* 3.

Ben-Yair, R., and Kalcheim, C. (2005). Lineage analysis of the avian dermomyotome sheet reveals the existence of single cells with both dermal and muscle progenitor fates. *Development* 132**,** 689-701.

Bertrand, S., Fuentealba, J., Aze, A., Hudson, C., Yasuo, H., Torrejon, M., Escriva, H., and Marcellini, S. (2013). A dynamic history of gene duplications and losses characterizes the evolution of the SPARC family in eumetazoans. *Proc Biol Sci* 280**,** 20122963.

Devoto, S.H., Stoiber, W., Hammond, C.L., Steinbacher, P., Haslett, J.R., Barresi, M.J.F., Patterson, S.E., Adiarte, E.G., and Hughes, S.M. (2006). Generality of vertebrate developmental patterns: evidence for a dermomyotome in fish. *Evolution & Development* 8**,** 101-110.

Dumont, E., Rallière, C., and Rescan, P.-Y. (2008). Identification of novel genes including Dermo-1, a marker of dermal differentiation, expressed in trout somitic external cells. *Journal of Experimental Biology* 211**,** 1163-1168.

Füchtbauer, E.M. (1995). Expression of M-twist during postimplantation development of the mouse. *Dev Dyn* 204**,** 316-322.

Gostling, N.J., and Shimeld, S.M. (2003). Protochordate Zic genes define primitive somite compartments and highlight molecular changes underlying neural crest evolution. *Evol Dev* 5**,** 136-144.

Goulding, M., Lumsden, A., and Paquette, A.J. (1994). Regulation of Pax-3 expression in the dermomyotome and its role in muscle development. *Development* 120**,** 957-971.

Grimaldi, A., Tettamanti, G., Martin, B.L., Gaffield, W., Pownall, M.E., and Hughes, S.M. (2004). Hedgehog regulation of superficial slow muscle fibres in Xenopus and the evolution of tetrapod trunk myogenesis. *Development* 131**,** 3249-3262.

Hammond, C.L., Hinits, Y., Osborn, D.P.S., Minchin, J.E.N., Tettamanti, G., and Hughes, S.M. (2007). Signals and myogenic regulatory factors restrict pax3 and pax7 expression to dermomyotome-like tissue in zebrafish. *Developmental Biology* 302**,** 504-521.

Himeda, C.L., Barro, M.V., and Emerson, C.P., Jr. (2013). Pax3 synergizes with Gli2 and Zic1 in transactivating the Myf5 epaxial somite enhancer. *Dev Biol* 383**,** 7-14.

Holland, L.Z., Pace, D.A., Blink, M.L., Kene, M., and Holland, N.D. (1995). Sequence and expression of amphioxus alkali myosin light chain (AmphiMLC-alk) throughout development: implications for vertebrate myogenesis. *Dev Biol* 171**,** 665-676.

Holland, L.Z., Schubert, M., Kozmik, Z., and Holland, N.D. (1999). AmphiPax3/7, an amphioxus paired box gene: insights into chordate myogenesis, neurogenesis, and the possible evolutionary precursor of definitive vertebrate neural crest. *Evol Dev* 1**,** 153-165.

Hornik, C., Krishan, K., Yusuf, F., Scaal, M., and Brand-Saberi, B. (2005). cDermo-1 misexpression induces dense dermis, feathers, and scales. *Dev Biol* 277**,** 42-50.

Horst, D., Ustanina, S., Sergi, C., Mikuz, G., Juergens, H., Braun, T., and Vorobyov, E. (2006). Comparative expression analysis of Pax3 and Pax7 during mouse myogenesis. *Int J Dev Biol* 50**,** 47-54.

Jostes, B., Walther, C., and Gruss, P. (1990). The murine paired box gene, Pax7, is expressed specifically during the development of the nervous and muscular system. *Mechanisms of Development* 33**,** 27-37.

Kozmik, Z., Holland, N.D., Kreslova, J., Oliveri, D., Schubert, M., Jonasova, K., Holland, L.Z., Pestarino, M., Benes, V., and Candiani, S. (2007). Pax–Six–Eya–Dach network during amphioxus development: Conservation in vitro but context specificity in vivo. *Developmental Biology* 306**,** 143-159.

Kusakabe, R., and Kuratani, S. (2005). Evolution and developmental patterning of the vertebrate skeletal muscles: perspectives from the lamprey. *Dev Dyn* 234**,** 824-834.

Li, L., Cserjesi, P., and Olson, E.N. (1995). Dermo-1: A Novel Twist-Related bHLH Protein Expressed in the Developing Dermis. *Developmental Biology* 172**,** 280-292.

Lu, T.-M., Luo, Y.-J., and Yu, J.-K. (2012). BMP and Delta/Notch signaling control the development of amphioxus epidermal sensory neurons: insights into the evolution of the peripheral sensory system. *Development* 139**,** 2020-2030.

Maroto, M., Reshef, R., Munsterberg, A.E., Koester, S., Goulding, M., and Lassar, A.B. (1997). Ectopic Pax-3 activates MyoD and Myf-5 expression in embryonic mesoderm and neural tissue. *Cell* 89**,** 139-148.

Mcmahon, A.R., and Merzdorf, C.S. (2010). Expression of the zic1, zic2, zic3, and zic4 genes in early chick embryos. *BMC Research Notes* 3**,** 167.

Meulemans, D., and Bronner-Fraser, M. (2007). Insights from Amphioxus into the Evolution of Vertebrate Cartilage. *PLOS ONE* 2**,** e787.

Nagai, T., Aruga, J., Takada, S., Günther, T., Spörle, R., Schughart, K., and Mikoshiba, K. (1997). The Expression of the MouseZic1, Zic2,andZic3Gene Suggests an Essential Role forZicGenes in Body Pattern Formation. *Developmental Biology* 182**,** 299-313.

Onimaru, K., Shoguchi, E., Kuratani, S., and Tanaka, M. (2011). Development and evolution of the lateral plate mesoderm: Comparative analysis of amphioxus and lamprey with implications for the acquisition of paired fins. *Developmental Biology* 359**,** 124-136.

Ota, K.G., Fujimoto, S., Oisi, Y., and Kuratani, S. (2011). Identification of vertebra-like elements and their possible differentiation from sclerotomes in the hagfish. *Nat Commun* 2**,** 373.

Pan, H., Gustafsson, M.K., Aruga, J., Tiedken, J.J., Chen, J.C., and Emerson, C.P., Jr. (2011). A role for Zic1 and Zic2 in Myf5 regulation and somite myogenesis. *Dev Biol* 351**,** 120-127.

Relaix, F., Rocancourt, D., Mansouri, A., and Buckingham, M. (2004). Divergent functions of murine Pax3 and Pax7 in limb muscle development. *Genes & development* 18**,** 1088-1105.

Rohr, K.B., Schulte-Merker, S., and Tautz, D. (1999). Zebrafish zic1 expression in brain and somites is affected by BMP and Hedgehog signalling. *Mechanisms of Development* 85**,** 147-159.

Scaal, M., Fuchtbauer, E.M., and Brand-Saberi, B. (2001). cDermo-1 expression indicates a role in avian skin development. *Anat Embryol (Berl)* 203**,** 1-7.

Schubert, M., Holland, L.Z., Stokes, M.D., and Holland, N.D. (2001). Three Amphioxus Wnt Genes (AmphiWnt3, AmphiWnt5, and AmphiWnt6) Associated with the Tail Bud: the Evolution of Somitogenesis in Chordates. *Developmental Biology* 240**,** 262-273.

Schubert, M., Meulemans, D., Bronner-Fraser, M., Holland, L.Z., and Holland, N.D. (2003). Differential mesodermal expression of two amphioxus MyoD family members (AmphiMRF1 and AmphiMRF2). *Gene Expr Patterns* 3**,** 199-202.

Stellabotte, F., Dobbs-Mcauliffe, B., Fernández, D.A., Feng, X., and Devoto, S.H. (2007). Dynamic somite cell rearrangements lead to distinct waves of myotome growth. *Development* 134**,** 1253-1257.

Sun Rhodes, L.S., and Merzdorf, C.S. (2006). The zic1 gene is expressed in chick somites but not in migratory neural crest. *Gene Expression Patterns* 6**,** 539-545.

Williams, B.A., and Ordahl, C.P. (1994). Pax-3 expression in segmental mesoderm marks early stages in myogenic cell specification. *Development* 120**,** 785-796.

Aldea, D., Leon, A., Bertrand, S., and Escriva, H. (2015). Expression of Fox genes in the cephalochordate Branchiostoma lanceolatum. *Frontiers in Ecology and Evolution* 3.

Ben-Yair, R., and Kalcheim, C. (2005). Lineage analysis of the avian dermomyotome sheet reveals the existence of single cells with both dermal and muscle progenitor fates. *Development* 132**,** 689-701.

Bertrand, S., Fuentealba, J., Aze, A., Hudson, C., Yasuo, H., Torrejon, M., Escriva, H., and Marcellini, S. (2013). A dynamic history of gene duplications and losses characterizes the evolution of the SPARC family in eumetazoans. *Proc Biol Sci* 280**,** 20122963.

Devoto, S.H., Stoiber, W., Hammond, C.L., Steinbacher, P., Haslett, J.R., Barresi, M.J.F., Patterson, S.E., Adiarte, E.G., and Hughes, S.M. (2006). Generality of vertebrate developmental patterns: evidence for a dermomyotome in fish. *Evolution & Development* 8**,** 101-110.

Dumont, E., Rallière, C., and Rescan, P.-Y. (2008). Identification of novel genes including Dermo-1, a marker of dermal differentiation, expressed in trout somitic external cells. *Journal of Experimental Biology* 211**,** 1163-1168.

Edgar, R.C. (2004). MUSCLE: multiple sequence alignment with high accuracy and high throughput. *Nucleic acids research* 32**,** 1792-1797.

Füchtbauer, E.M. (1995). Expression of M-twist during postimplantation development of the mouse. *Dev Dyn* 204**,** 316-322.

Gostling, N.J., and Shimeld, S.M. (2003). Protochordate Zic genes define primitive somite compartments and highlight molecular changes underlying neural crest evolution. *Evol Dev* 5**,** 136-144.

Goulding, M., Lumsden, A., and Paquette, A.J. (1994). Regulation of Pax-3 expression in the dermomyotome and its role in muscle development. *Development* 120**,** 957-971.

Grimaldi, A., Tettamanti, G., Martin, B.L., Gaffield, W., Pownall, M.E., and Hughes, S.M. (2004). Hedgehog regulation of superficial slow muscle fibres in Xenopus and the evolution of tetrapod trunk myogenesis. *Development* 131**,** 3249-3262.

Hammond, C.L., Hinits, Y., Osborn, D.P.S., Minchin, J.E.N., Tettamanti, G., and Hughes, S.M. (2007). Signals and myogenic regulatory factors restrict pax3 and pax7 expression to dermomyotome-like tissue in zebrafish. *Developmental Biology* 302**,** 504-521.

Himeda, C.L., Barro, M.V., and Emerson, C.P., Jr. (2013). Pax3 synergizes with Gli2 and Zic1 in transactivating the Myf5 epaxial somite enhancer. *Dev Biol* 383**,** 7-14.

Holland, L.Z., Pace, D.A., Blink, M.L., Kene, M., and Holland, N.D. (1995). Sequence and expression of amphioxus alkali myosin light chain (AmphiMLC-alk) throughout development: implications for vertebrate myogenesis. *Dev Biol* 171**,** 665-676.

Holland, L.Z., Schubert, M., Kozmik, Z., and Holland, N.D. (1999). AmphiPax3/7, an amphioxus paired box gene: insights into chordate myogenesis, neurogenesis, and the possible evolutionary precursor of definitive vertebrate neural crest. *Evol Dev* 1**,** 153-165.

Hornik, C., Krishan, K., Yusuf, F., Scaal, M., and Brand-Saberi, B. (2005). cDermo-1 misexpression induces dense dermis, feathers, and scales. *Dev Biol* 277**,** 42-50.

Horst, D., Ustanina, S., Sergi, C., Mikuz, G., Juergens, H., Braun, T., and Vorobyov, E. (2006). Comparative expression analysis of Pax3 and Pax7 during mouse myogenesis. *Int J Dev Biol* 50**,** 47-54.

Jostes, B., Walther, C., and Gruss, P. (1990). The murine paired box gene, Pax7, is expressed specifically during the development of the nervous and muscular system. *Mechanisms of Development* 33**,** 27-37.

Kozmik, Z., Holland, N.D., Kreslova, J., Oliveri, D., Schubert, M., Jonasova, K., Holland, L.Z., Pestarino, M., Benes, V., and Candiani, S. (2007). Pax–Six–Eya–Dach network during amphioxus development: Conservation in vitro but context specificity in vivo. *Developmental Biology* 306**,** 143-159.

Kusakabe, R., and Kuratani, S. (2005). Evolution and developmental patterning of the vertebrate skeletal muscles: perspectives from the lamprey. *Dev Dyn* 234**,** 824-834.

Letunic, I., Doerks, T., and Bork, P. (2014). SMART: recent updates, new developments and status in 2015. *Nucleic Acids Research* 43**,** D257-D260.

Li, L., Cserjesi, P., and Olson, E.N. (1995). Dermo-1: A Novel Twist-Related bHLH Protein Expressed in the Developing Dermis. *Developmental Biology* 172**,** 280-292.

Lu, T.-M., Luo, Y.-J., and Yu, J.-K. (2012). BMP and Delta/Notch signaling control the development of amphioxus epidermal sensory neurons: insights into the evolution of the peripheral sensory system. *Development* 139**,** 2020-2030.

Maroto, M., Reshef, R., Munsterberg, A.E., Koester, S., Goulding, M., and Lassar, A.B. (1997). Ectopic Pax-3 activates MyoD and Myf-5 expression in embryonic mesoderm and neural tissue. *Cell* 89**,** 139-148.

Mcmahon, A.R., and Merzdorf, C.S. (2010). Expression of the zic1, zic2, zic3, and zic4 genes in early chick embryos. *BMC Research Notes* 3**,** 167.

Meulemans, D., and Bronner-Fraser, M. (2007). Insights from Amphioxus into the Evolution of Vertebrate Cartilage. *PLOS ONE* 2**,** e787.

Nagai, T., Aruga, J., Takada, S., Günther, T., Spörle, R., Schughart, K., and Mikoshiba, K. (1997). The Expression of the MouseZic1, Zic2,andZic3Gene Suggests an Essential Role forZicGenes in Body Pattern Formation. *Developmental Biology* 182**,** 299-313.

Onimaru, K., Shoguchi, E., Kuratani, S., and Tanaka, M. (2011). Development and evolution of the lateral plate mesoderm: Comparative analysis of amphioxus and lamprey with implications for the acquisition of paired fins. *Developmental Biology* 359**,** 124-136.

Ota, K.G., Fujimoto, S., Oisi, Y., and Kuratani, S. (2011). Identification of vertebra-like elements and their possible differentiation from sclerotomes in the hagfish. *Nat Commun* 2**,** 373.

Pan, H., Gustafsson, M.K., Aruga, J., Tiedken, J.J., Chen, J.C., and Emerson, C.P., Jr. (2011). A role for Zic1 and Zic2 in Myf5 regulation and somite myogenesis. *Dev Biol* 351**,** 120-127.

Relaix, F., Rocancourt, D., Mansouri, A., and Buckingham, M. (2004). Divergent functions of murine Pax3 and Pax7 in limb muscle development. *Genes & development* 18**,** 1088-1105.

Rohr, K.B., Schulte-Merker, S., and Tautz, D. (1999). Zebrafish zic1 expression in brain and somites is affected by BMP and Hedgehog signalling. *Mechanisms of Development* 85**,** 147-159.

Scaal, M., Fuchtbauer, E.M., and Brand-Saberi, B. (2001). cDermo-1 expression indicates a role in avian skin development. *Anat Embryol (Berl)* 203**,** 1-7.

Schubert, M., Holland, L.Z., Stokes, M.D., and Holland, N.D. (2001). Three Amphioxus Wnt Genes (AmphiWnt3, AmphiWnt5, and AmphiWnt6) Associated with the Tail Bud: the Evolution of Somitogenesis in Chordates. *Developmental Biology* 240**,** 262-273.

Schubert, M., Meulemans, D., Bronner-Fraser, M., Holland, L.Z., and Holland, N.D. (2003). Differential mesodermal expression of two amphioxus MyoD family members (AmphiMRF1 and AmphiMRF2). *Gene Expr Patterns* 3**,** 199-202.

Stamatakis, A. (2014). RAxML version 8: a tool for phylogenetic analysis and post-analysis of large phylogenies. *Bioinformatics* 30**,** 1312-1313.
Stecher, G., Tamura, K., and Kumar, S. (2020). Molecular Evolutionary Genetics Analysis (MEGA) for macOS. *Mol Biol Evol* 37**,** 1237-1239.
Stellabotte, F., Dobbs-Mcauliffe, B., Fernández, D.A., Feng, X., and Devoto, S.H. (2007). Dynamic somite cell rearrangements lead to distinct waves of myotome growth. *Development* 134**,** 1253-1257.
Sun Rhodes, L.S., and Merzdorf, C.S. (2006). The zic1 gene is expressed in chick somites but not in migratory neural crest. *Gene Expression Patterns* 6**,** 539-545.
Williams, B.A., and Ordahl, C.P. (1994). Pax-3 expression in segmental mesoderm marks early stages in myogenic cell specification. *Development* 120**,** 785-796.
